# Synthesis of (2*S,*3*R*)-3-amino-2-hydroxydecanoic acid and its enantiomer: a non-proteinogenic amino acid segment of the linear pentapeptide microginin

**DOI:** 10.3762/bjoc.10.59

**Published:** 2014-03-17

**Authors:** Rajendra S Rohokale, Dilip D Dhavale

**Affiliations:** 1 Department of Chemistry, Garware Research Centre, University of Pune, Pune - 411 007, India

**Keywords:** AHDA, carbohydrate, chiron approach, enantioselective, natural products, non-proteinogenic amino acid

## Abstract

A directed manipulation of the functional groups at C3 and C4 of D-glucose was demonstrated to synthesize naturally occurring (2*S,*3*R*)-α-hydroxy-β-aminodecanoic acid (AHDA, **2a**) and its enantiomer **2b**. The enantiomer of **2a** is the N-terminal part of the natural linear pentapeptide microginin, which is used as an antihypertensive agent.

## Introduction

**Figure 1:**
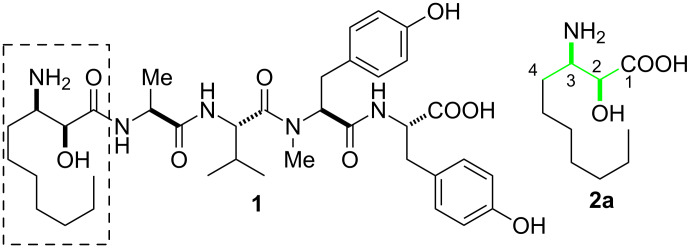
Microginin (**1**) and (2*S*,3*R*)-AHDA (**2a**).

Microginin **1** (Figure 1), isolated from the cyanobacterium *Microcystis aeruginosa,* is a linear pentapeptide consisting of L-Tyr-L-*N*-Me-Tyr-L-Val-L-Ala and (2*S*,3*R*)-α-hydroxy-β-aminodecanoic acid ((2*S*,3*R*)-AHDA, **2a**) [1]. Microginin is used as a hypertensive agent based on its biological activity against angiotensin converting enzyme, which is responsible for the vasoconstriction of blood vessels [2–4]. Amongst different amino acids present in microginin, (2*S*,3*R*)-AHDA (**2a**) is a non-proteinogenic natural amino acid attached at the N-terminal part of the peptide chain. The α-hydroxy-β-amino acid fragment in AHDA **2a** is also present in linear peptides such as bestatin and valinoctin [5–8], which are isolated from the same species. In addition, the chiral α-hydroxy-β-amino acid constituent is an important component of protein kinase inhibitor compounds like balanol and the anticancer drug taxol [9].

Due to the biological importance of (2*S*,3*R*)-AHDA, the enantioselective synthesis of its chiral core is a challenge task. This fact led to several approaches for the stereoselective synthesis of (2*S*,3*R*)-AHDA including (i) the enantioselective introduction of amino- and hydroxy-groups to olefinic acid by either asymmetric epoxidation, dihydroxylation or aminohydroxylation [10–15], (ii) the asymmetric synthesis of β-lactams by a Staudinger reaction between ketene and imine to give the corresponding amino acids [16], and (iii) the Lewis acid catalyzed multicomponent condensation reactions of aldehyde, an amine and ketene silyl acetal derivatives to get the vicinal hydroxylamino acids [17]. In addition, a few strategies employ a chiral pool approach. For example, Wee et al. utilized the zinc-silver-mediated reductive elimination of α-D-lyxofuranosyl phenylsulfone to get (4*S*,5*S*)-4-formyl-5-vinyl-2-oxazolidone, which was converted into **2a** [18]. Merrer and co-workers used D-isoascorbic acid, which was transformed via (2*R*)-amino-1,3,4-triol to **2a** [19]. Bergmeier et al. synthesized a chiral allyl alcohol from D-mannitol, which is converted to the azidoformate and thermally cyclized to a bicyclic aziridine. The opening of the aziridine with organocuprate led to a corresponding chiral hydroxylated amino acid core [20]. Although a number of chiron approaches are known [21], there is no report from D-glucose towards the synthesis of (2*S*,3*R*)-AHDA (**2a**) and its enantiomer (2*R*,3*S*)-AHDA (**2b**). As a part of our continuous interest in the synthesis of chiral amino acids [22–23] and their utility in the synthesis of iminosugars [24–29], we report here an efficient and practical approach for the synthesis of both enantiomers of AHDA (**2a** and **2b**) from the same precursor D-glucose by simple manipulation of the functional groups.

**Scheme 1:**
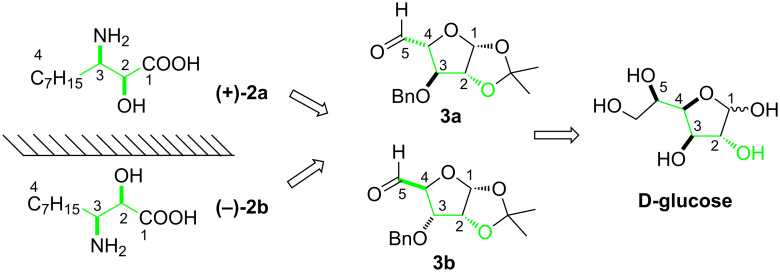
Retrosynthetic analysis of AHDA.

We visualized that the structural and the stereochemical symmetry of both enantiomers (**2a**/**2b**) is present in D-glucose. The C1-carboxyl carbon atom of **2a** is present at the C2 of the D-glucose, and the C4 carbon atom with an alkyl chain in **2a** could be built on C5 of the D-glucose (Scheme 1). The required relative stereochemistry of the vicinal hydroxyamino functionality in **2a** at C2 and C3 is embedded at the C3 and C4 of D-glucose, respectively, and needs to be manipulated by usual functional group transformations. Thus, for the synthesis of enantiomers **2a** and **2b** the corresponding sugar precursors were found to be suitably protected β-L-arabino-pentodialdo-1,4-furanose **3a** [30–31] and α-D-ribo-pentodialdo-1,4-furanose **3b** [32]. There exists a distinct possibility to synthesize these chiron synthons **3a** and **3b** from the easily available and cheap starting material D-glucose. Our results of the synthesis of both enantiomers **2a** and **2b** are described herein.

## Results and Discussion

**Scheme 2:**
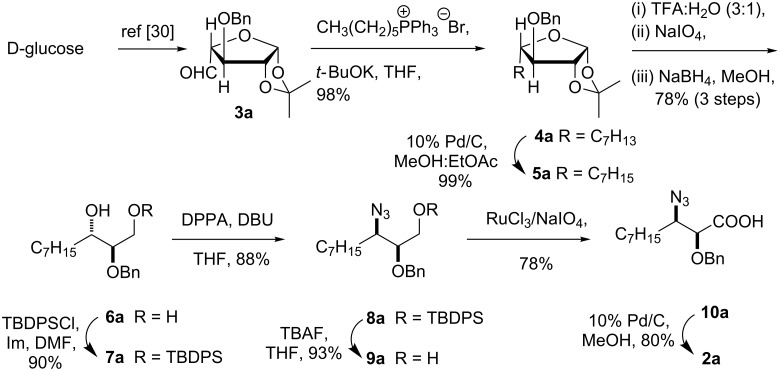
Synthesis of AHDA **2a**.

As reported earlier, D-glucose was converted to the 3-*O*-benzyl-1,2-*O*-isopropylidene-β-L-arabino-pentodialdo-1,4-furanose (**3a**) in 72% yield (Scheme 2) [30]. While targeting the synthesis of **2a**, the Wittig olefination of **3a** with *n*-hexyltriphenylphosphonium bromide and *t*-BuOK gave olefin **4a** as a diasteromeric mixture of *Z* and *E-*isomers in the ratio 9.5:0.5 as shown by ^1^H NMR of the crude product. The catalytic hydrogenation of alkene **4a** with 10% Pd/C in methanol:ethyl acetate (3:2) at balloon pressure gave 4-heptyl-L-threose derivative **5a** as a viscous oil in 99% yield [33]. Removal of the 1,2-acetonide group with TFA–water in **5a** provided an anomeric mixture of the hemiacetal, which was directly subjected to oxidative cleavage by using sodium metaperiodate in acetone–water (to cleave the anomeric carbon) followed by a treatment with sodium borohydride to give triol **6a** as a viscous oil in 78% overall yield in three steps [34]. The primary hydroxy group of triol **6a** was selectively monosilylated with *t*-butyldiphenylsilyl chloride to give **7a**. Subsequently, the secondary hydroxy group in **7a** was converted to azido derivative **8a** with an inversion of the configuration by using diphenylphosphoryl azide in the presence of DBU in 88% yield [35]. Cleavage of the silyl functionality in **8a** with *n*-tetrabutylammonium fluoride offered azido alcohol **9a** as a viscous oil. The azido alcohol **9a** was oxidized to the corresponding acid by using RuCl_3_·3H_2_O/NaIO_4_ to give **10a** [36].

Finally, cleavage of the 2-*O*-benzyl ether and reduction of the 3-azido group to the corresponding amine in one step with 10% Pd/C in methanol provided (−)-α-hydroxy-β-aminodecanoic acid (AHDA, **2a**) in 80% yield as a white solid. The spectral and analytical data of **2a** was found to be in good agreement with published data ([α]_D_^25^ +5.6 (*c* 0.51, 1 M HCl). [α]_D_^22^ +7.3 (*c* 0.37, 1 M HCl)) [18].

**Scheme 3:**
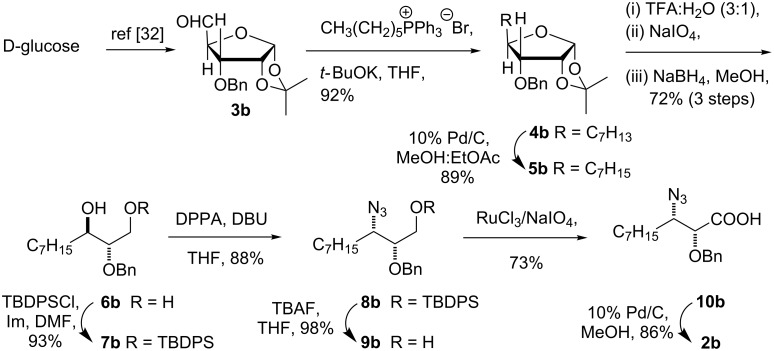
Synthesis of *ent*-AHDA **2b**.

The synthesis of AHDA enantiomer **2b** was accomplished starting from 3-*O*-benzyl-1,2-*O*-isopropylidene-α-D-ribo-pentodialdo-1,4-furanose (**3b**) which was obtained from D-glucose in good yield as reported earlier [33]. Thus, the Wittig reaction of **3b** followed by hydrogenation (10% Pd/C) gave 4-heptyl-D-threose derivative **5b** (Scheme 3). Hydrolysis of 1,2-*O*-isopropylidene (TFA:H_2_O) followed by oxidative cleavage of the hemiacetal with NaIO_4_ and reduction with NaBH_4_ gave triol **6b**,which was monosilylated with TBDPSCl to give **7b**. Conversion of the secondary hydroxy group in **7b** to azide **8b** according to the Mitsunobu protocol, and deprotection followed by oxidation of the primary hydroxy group gave azido acid **10b**. Finally, hydrogenolysis of the benzyl group and reduction of the azido group by using 10% Pd/C, in one pot, gave **2b** in 30.1% overall yield from **3b**. The spectral and analytical data was found to be in good agreement with reported data ([α]_D_^30^ −5.1(*c* 0.51, 1 M HCl). [α]_D_^30^ −6.2 (*c* 0.4, 1 M HCl)) [21].

## Conclusion

In conclusion, we demonstrated a practical approach for the synthesis of both enantiomers of AHDA (**2a** and **2b**) to obtain the stereochemistry required for the α-hydroxy-β-amino acid. Our method starts from D-glucose by an easy manipulation of its functional groups at C3 and C4. In addition, the chiral core (α-hydroxy-β-amino acid) in **2a** is present in several biologically active compounds such as taxol, balanol and bestatin. Therefore, this methodology could be potentially exploited for the synthesis of the chiral segment of these compounds.

## Supporting Information

File 1 Experimental procedures.

File 2 Copies of NMR spectra.

## References

[1] Okino T, Matsuda H, Murakami M, Yamaguchi K (1993). Tetrahedron Lett.

[2] Wyvratt M J, Patchett A A (1985). Med Res Rev.

[3] Moore R E, Banarjee S, Bomemann V, Caplan F R, Chen J L, Corley D G, Larsen L K, Moore B S, Patterson G M L, Paul V J (1989). Pure Appl Chem.

[R4] 4Carmichael W. W. *Handbook of Natural Toxins*; Tu, A. T.; Ed.; Marcel Dekker: New York, 1988; pp. 121 ff.

[5] Umezawa H, Aoyagi T, Suda H, Hamada M, Takeuchi T (1976). J Antibiot.

[6] Nakamura H, Suda H, Takita T, Aoyagi T, Umezawa H (1976). J Antibiot.

[7] Sekizawa R, Iinuma H, Muraoka Y, Naganawa H, Kinoshita N, Nakamura H, Hamada M, Takeuchi T, Umezawa K (1996). J Nat Prod.

[8] Tsuda M, Muraoka Y, Takeuchi T (1996). J Antibiot.

[9] Nicolau K C, Dai W-M, Guy R K (1994). Angew Chem, Int Ed.

[10] Bunnage M E, Burke A J, Davies S G, Goodwin C J (1994). Tetrahedron: Asymmetry.

[11] Li G, Chang H-T, Sharpless K B (1996). Angew Chem, Int Ed.

[12] Chandrasekhar S, Mohapatra S, Yadav J S (1997). Tetrahedron.

[13] Sugimura H, Miura M, Yamada N (1997). Tetrahedron: Asymmetry.

[14] Righi G, Chionne A, D’Achille R, Bonini C (1997). Tetrahedron: Asymmetry.

[15] Jeffords C W, Mcnulty J, Lu Z H, Wang J B (1996). Helv Chim Acta.

[16] Ha H J, Ahn Y G, Woo J S, Lee G S, Lee W K (2001). Bull Chem Soc Jpn.

[17] Gassa F, Contini A, Fontana G, Pellegrino S, Gelmi M L (2010). J Org Chem.

[18] Wee A G H, McLeod D D (2003). J Org Chem.

[19] Tuch A, Saniere M, Merrer Y L, Depezay J-C (1996). Tetrahedron: Asymmetry.

[20] Bergmeier S C, Stanchina D M (1999). J Org Chem.

[21] Shirode N M, Deshmukh A R A S (2006). Tetrahedron.

[22] Kalamkar N B, Kasture V M, Dhavale D D (2010). Tetrahedron Lett.

[23] Kalamkar N B, Kasture V M, Dhavale D D (2008). J Org Chem.

[24] Dhavale D D, Markad S D, Karanjule N S, Prakasha Reddy J (2004). J Org Chem.

[25] Karanjule N S, Markad S D, Dhavale D D (2006). J Org Chem.

[26] Karanjule N S, Markad S D, Shinde V S, Dhavale D D (2006). J Org Chem.

[27] Dhavale D D, Ajish Kumar K S, Chaudhari V D, Sharma T, Sabharwal S G, Prakasha R (2005). Org Biomol Chem.

[28] Ajish Kumar K S, Chaudhari V D, Puranik V G, Dhavale D D (2007). Eur J Org Chem.

[29] Pawar N J, Parihar V, Chavan S, Joshi R, Joshi P V, Sabharwal S G, Puranik V G, Dhavale D D (2012). J Org Chem.

[30] Sato K-i, Akai S, Sakuma M, Kojima M, Suzuki K-j (2003). Tetrahedron Lett.

[31] Hanessian S (1983). Total Synthesis of Natural Products: The "Chiron" Approach.

[32] Patil N T, John S, Sabharwal S G, Dhavale D D (2002). Bioorg Med Chem.

[33] Bindra J, Grodski A (1978). J Org Chem.

[34] Mane R S, Ajish Kumar K S, Dhavale D D (2008). J Org Chem.

[35] Thompson A S, Humphrey G R, DeMarco A M, Marthe D J, Grabowaski E J J (1993). J Org Chem.

[36] Carlsen P J H, Katsuki T, Martin V S, Sharpless K B (1981). J Org Chem.

