# Supplementary material for: Synthesis of (2S,3R)-3-amino-2-hydroxydecanoic acid and its enantiomer: a non-proteinogenic amino acid segment of the linear pentapeptide microginin
Source: Beilstein J Org Chem. 2014 Mar 17;10:667–71. doi: 10.3762/bjoc.10.59 (PMC3999816; doi:10.3762/bjoc.10.59)
Supplement: File 1 — Experimental procedures. [file Beilstein_J_Org_Chem-10-667-s001.pdf]

Supporting Information

for

Synthesis of (2*S*,3*R*)-3-amino-2-hydroxydecanoic acid and its enantiomer: a non-proteinogenic amino acid segment of the linear pentapeptide microginin

Rajendra S. Rohokale and Dilip D. Dhavale*

Address: Department of Chemistry, Garware Research Centre, University of Pune, Pune - 411 007, India

Email: Dilip D. Dhavale - ddd@chem.unipune.ac.in

* Corresponding author

Experimental procedures

General experimental methods:

Melting points were recorded with a Thomas Hoover Capillary melting point apparatus and are uncorrected. IR spectra were recorded with a Shimadzu FTIR-8400 spectrometer as a thin film or by using KBr pellets and are expressed in cm^{-1}. ^{1}H (300 MHz) and ^{13}C (75 MHz) NMR spectra were recorded with a Varian Mercury instrument by using $CDCl_3$, D_2O or DMSO-d_6 as solvent. Chemical shifts are reported in δ unit (ppm) with reference to TMS (Me_4Si) as an internal standard. High-resolution mass spectra were obtained from a Micro Mass ESI-TOF MS spectrometer. Optical rotations were measured by using a JASCO P-1020 digital polarimeter with sodium light (589.3 nm) at 25–35 °C. Thin-layer chromatography was performed on pre-coated plates (0.25 mm, silica gel 60 F254). Visualization was made

by absorption of UV light or by thermal development after spraying with 3.5% solution of 2,4-dinitrophenylhydrazine in ethanol/H_2SO_4 and with basic aqueous potassium permanganate solution. Column chromatography was carried out with silica gel (100–200 mesh). Unless not mentioned the reactions were carried out in oven-dried glasswares under dry N_2. Acetone, dichloromethane, THF, acetonitrile, and methanol were purified and dried before use. Distilled *n*-hexane, ethyl acetate, chloroform, and methanol were used for column chromatography. After quenching the reaction with water, the work-up involves washing of the combined organic layer with water, brine, drying over anhydrous sodium sulfate, and evaporation of the solvent at reduced pressure by using a rotary evaporator.

Experimental procedures:

Compound 4a. To a stirred solution of the *n*-hexyltriphenylphosphonium bromide (8.63 g, 20.23 mmol) in dry THF (30 mL) at −20 °C was added potassium *tert*-butoxide (2.26 g, 20.23 mmol), the change in colour from white to yellowish orange was noticed as the reaction progress. The reaction mixture was stirred for 40 min at room temperature. A solution **3a** (3.57 g, 13.48 mmol) in THF (15 mL) was added dropwise at −20 °C and allowed to warm to room temperature. The reaction mixture was quenched after 6 h with saturated aqueous solution of NH_4Cl (5 mL). The solvent was evaporated under reduced pressure, and the residue was extracted in EtOAc (20 mL × 3), usual work-up followed by purification by flash column chromatography (pet ether/ethyl acetate 8:2) afforded an inseparable diastereomeric mixture of *E/Z*-isomers of **4a** (4.5g, 98 %) as colorless viscous oil. Data for **4a**: R_f = 0.8 (pet ether/ethyl acetate 6:4); IR (neat) ν_{max}: 2108, 1635 cm^{-1}; ^1H NMR (300 MHz, $CDCl_3$) NMR δ 0.87 (t, *J* = 6.6 Hz, 3H), 1.22-1.5 (m, 11H), 1.54 (s, 3H) 1.9-2.18 (m, 2H), 3.88 (d, *J* = 3.2 Hz, 1H), 4.60 (ABq, *J* = 11.6 Hz, 2H), 4.66 (d, *J* = 3.8 Hz, 1H), 4.88 (dd, *J* = 6.4, 3.2 Hz, 1H), 5.48-5.7 (m, 2H), 5.92 (dd, *J* = 3.8 Hz, 1H), δ 7.4 (m, 5H). ^{13}C NMR (75 MHz, $CDCl_3$) NMR δ 13.9, 22.5, 26.4, 27.0, 27.5, 29.2, 31.4, 71.9, 80.6, 85.2, 87.2, 105.5,

112.8, 127.6, 127.6, 128.0, 128.4, 133.9, 137.3. HRMS (ESI TOF) *m*/*z* calcd. for $C_{21}H_{30}O_4Na$ $[M + Na]^+$ 369.2144, found 369.2142.

(The ^1H and ^{13}C NMR spectrum of compound **4a** showed additional signals corresponding to the minor *E*-isomer < 5%).

Compound 5a. Compound **4a** (2.0 g, 5.74 mmol) was dissolved in MeOH/EtOAc (3:2) (20 mL) and 10% Pd/C (80 mg) was added. The mixture was hydrogenated at balloon pressure of H_2 gas for 2 h at room temperature. The catalyst was removed by filtration through celite and washed with EtOAc. The solvent was removed under reduced pressure and the crude product was purified by flash column chromatography on silica gel (pet ether/ethyl acetate 8:2 as eluent) to afford compound **5a**; (2.0 g, 99%) as viscous oil. R_f = 0.7 (pet ether/ethyl acetate 9.9:0.1); $[\alpha]_D^{27}$ -22.5 (*c* 12, $CHCl_3$); IR (neat) ν_{max}: 2924, 1460, 1016 cm^{-1}; ^1H NMR (300 MHz, $CDCl_3$): δ 0.87 (t, *J* = 6.6 Hz, 3H), 1.22-1.5 (m, 12H), 1.54-1.9 (m, 6H), 3.88 (d, *J* = 3.2 Hz, 1H), 4.60 (ABq, *J* = 11.4 Hz, 2H), 4.66 (d, *J* = 3.8 Hz, 1H), 4.88 (dd, *J* = 6.3, 3.1 Hz, 1H), 5.92 (dd, *J* = 3.8 Hz, 1H), δ 7.4 (m, 5H). ^{13}C NMR (75 MHz, $CDCl_3$): δ 14.0, 22.6, 25.5, 26.7, 29.1, 29.6, 31.7, 32.2, 72.0, 81.6, 103.8, 112.5, 127.9, 128.4, 128.4, 137.6. HRMS (ESI TOF) *m*/*z* calcd. for $C_{21}H_{32}O_4Na$ $[M + Na]^+$ 371.2198, found 371.2198.

Compound 6a. An ice-cold solution of **5a** (2.0 g, 5.74 mmol) in TFA/H_2O (20 mL, 3:2) was stirred for 15 min and at 25 °C for 4 h. Trifluoroacetic acid was co-evaporated with toluene at a rotary evaporator to furnish the hemiacetal as a thick liquid. To an ice-cold solution of the hemiacetal (1.57 g, 5.09 mmol) in acetone/water (20 mL, 5:1) was added sodium metaperiodate (1.6 g, 7.64 mmol) and the solution was stirred for 1.5 h at 25 °C. After completion of the reaction, the reaction mixture was quenched with 2 mL ethylene glycol. The solvent was evaporated on a rotary evaporator and the residue was extracted with EtOAc (15 mL × 3). The solvent was evaporated at reduced pressure. To an ice-cold solution of the crude product (1.2 g, 3.92 mmol) in THF/water (10 mL, 4:1) was added sodium borohydride

(0.29 g, 7.84 mmol) in two portions. The reaction mixture was stirred for 30 min and quenched by adding saturated aq NH_4Cl solution (5 mL). THF was evaporated under reduced pressure, extracted with chloroform (20 mL × 3) and concentrated, purification by column chromatography (pet ether/ethyl acetate 9:1) gave **6a** (0.85 g, 78% three steps) as a thick colourless liquid: R_f = 0.45 (pet ether/ethyl acetate 6:4); $[\alpha]_D^{33.2}$ -27.8, (*c* 0.34, $CHCl_3$); IR (neat) ν_{max}: 3389 (br), 1739, 1460 cm^{-1}; ^{1}H NMR (300 MHz, $CDCl_3$): δ 0.9 (t, *J* = 6.9 Hz, 3H), 1.2-1.7 (m, 12H), 1.75 (br, s, 1H, exch. with D_2O), 2.35 (br s, 1H, exch. with D_2O), 3.3-3.4 (m, 1H), 3.78-4.0 (m, 3H), 4.62 (s, 2H), 7.2-7.42 (m, 5H). ^{13}C NMR (75 MHz, $CDCl_3$): δ 14.0, 22.6, 25.9, 29.2, 29.5, 31.7, 32.8, 60.9, 71.8, 71.9, 81.3, 127.9, 127.9, 128.5, 137.9. HRMS (ESI TOF) *m/z* calcd. for $C_{17}H_{28}O_3Na$ $[M + Na]^+$ 303.1936; found 303.1935.

Compound 7a. To a solution of **6a** (0.7 g, 2.50 mmol) and imidazole (0.45 g, 7.50 mmol) in DMF (10 mL) at 0 °C was added a solution of TBDPSCl (0.618 g, 2.24 mmol) in DMF (2.5 mL). The reaction mixture was warmed to room temperature and stirred for 8 h. The reaction mixture was quenched with saturated aqueous NH_4Cl and extracted with EtOAc (30 mL × 3), usual work-up and purification on flash column chromatography (*n*-hexane/ethyl acetate 9:1) gave **7a** (1.28 g, 90%) as thick liquid. R_f = 0.9 (pet ether/ethyl acetate 8:2); $[\alpha]_D^{33.5}$ -22.1 (*c* 0.32, $CHCl_3$); IR (neat) ν_{max}: 3356 (br), 1658, 1427 cm^{-1}; ^{1}H NMR (300 MHz, $CDCl_3$): δ 0.83-1.5 (m, 22H , on exchange with D_2O showed 21H), 3.58-3.68 (m, 1H), 3.78 (dd, *J* = 10.9, 6.4 Hz, 1H), 3.86 (dd, *J* = 10.9 Hz, 1H), 3.87-3.95 (m, 1H), 4.64 (ABq, *J* = 11.8 Hz , 2H), 7.2-7.7 (m, 15H). ^{13}C NMR (75 MHz, $CDCl_3$): δ 14.1, 19.1, 19.4, 22.6, 25.4, 26.8, 27.1, 29.1, 29.6, 31.7, 33.1, 64.8, 72.8, 74.1, 83, 127.2, 127.4, 127.6, 128.2, 129.3, 129.5, 133.5, 133.7, 133.8, 134.0, 135.6, 136, 136.1, 139.1. HRMS (ESI TOF) *m/z* calcd. for $C_{33}H_{46}O_3SiNa$ $[M + Na]^+$ 541.3114; found 541.3116.

Compound 8a. To a solution of **7a** (1.2 g, 2.31 mmol) in THF (10 mL) was added a solution of diphenylphosporyl azide (0.99 mL, 4.63 mmol) and DBU at 0 °C. The reaction mixture

was stirred for 12 h at room temperature and quenched with water. Extraction with EtOAc (30 mL × 3), work-up and purification on column chromatography (*n*-hexane/ethyl acetate 9:1) gave **8a** (1.1 g, 88%) as thick liquid. R_f = 0.6 (pet ether/ethyl acetate 9:1); $[\alpha]_D^{33.5}$ +41.6 (*c* 0.46, $CHCl_3$); IR (neat) ν_{max}: 2102, 1600, 1462 cm^{-1}; ^1H NMR (300 MHz, $CDCl_3$): δ 0.9 (t, *J* = 6.6 Hz, 3H), 1.16-1.62 (m, 12H), 3.38-3.52 (m, 2H), 3.74-3.86 (m, 2H), 4.43 (d, *J* = 11.8Hz, 1H), 4.61 (d, *J* = 11.8Hz, 1H), 7.2-7.5 (m, 11H), 7.6-7.8 (m, 4H). ^{13}C NMR (75 MHz, $CDCl_3$): δ 14.0, 19.1, 19.4, 22.6, 25.8, 26.5, 26.8, 29.2, 29.6, 31.8, 32.6, 63.8, 68.2, 72.7, 81.1, 127.6, 127.7, 128.3, 129.9, 132.9, 133.1, 134.7, 135.5, 135.6, 138.4. HRMS (ESI TOF) *m*/*z* calcd. for $C_{33}H_{46}N_3O_2SiNa$ $[M + Na]^+$ 566.3179; found 566.3178.

Compound 9a. To a solution of **8a** (1.0 g, 1.84 mmol) in THF (7 mL) at 0 °C was added *n*-Bu_4NF (2.76 mL of a 1.0 M solution in THF, 2.76 mmol) dropwise over 5 min. The reaction mixture was stirred at 0 °C for 30 min and then diluted with EtOAc. The combined organic layer was with extracted with aqueous phase (30 mL × 3). Usual work-up and column chromatography (pet ether/ethyl acetate 9:1) offerded **9a** (0.548 g, 93%) as a colorless thick liquid. R_f = 0.2 (pet ether /ethyl acetate = 9/1); $[\alpha]_D^{33.6}$ +32.3 (*c* 0.41, $CHCl_3$); IR (neat) ν_{max}: 3350 (br) 2105, 1600, 1462 cm^{-1}; ^1H NMR (300 MHz, $CDCl_3$): δ 0.86 (t, *J* = 6.6 Hz, 3H), 1.18-1.62 (m, 12H), 1.78-1.86 (s, 1H exch. with D_2O), 3.3-3.52 (m, 2H), 3.65-3.68 (dd, *J* = 12, 4.8 Hz, 1H), 3.79-3.8 (dd, *J* = 11.9, 3.9 Hz, 1H), 4.64-4.7 (ABq, *J* = 1.7 Hz, 2H), δ 7.25- 7.5 (m, 5H). ^{13}C NMR (75 MHz, $CDCl_3$): δ 14.0, 22.5, 26.2, 29.0, 29.2, 30.2, 31.7, 61.5, 63.1, 73.2, 81.6, 127.91, 127.99, 128.5, 137.7. HRMS (ESI TOF) *m*/*z* calcd. for $C_{17}H_{27}N_3O_2Na$ $[M + Na]^+$ 328.2001; found 328.2000.

Compound 10a. To a solution of **9a** (0.400 mg, 1.31 mmol) in CCl_4 (1 mL), CH_3CN (1 mL), and water (1.5 mL) was added $NaIO_4$ (0.56 mg, 2.62 mmol) followed by $RuCl_3{\cdot}3H_2O$ (0.008 mg, 0.039 mmol). The reaction mixture was stirred vigorously for 15 h. The aqueous phase was extracted with CH_2Cl_2 (20 mL × 3). The combined organic layer was dried (Na_2SO_4),

filtered, and concentrated on rotary evaporator. The resulting crude material was diluted with EtOAc (30 mL), and a saturated aqueous solution of K_2CO_3 was added. This solution was extracted with EtOAc (30 mL × 3). The aqueous layer was acidified with 6 M HCl to pH 1 and extracted with EtOAc (30 mL × 3). The combined organic layer was dried (Na_2SO_4), filtered, and concentrated to afford acid **10a** (0.341 g, 78%) as a colorless oil. The acid was not subjected to further purification and was used immediately in the next reaction. R_f = 0.2 (*n*-hexane/ethyl acetate = 9/1); $[\alpha]_D^{33.6}$ +27.8 (*c* 0.51, $CHCl_3$); IR (neat) ν_{max}: 3350 (br) 2105, 1600, 1462 cm^{-1}; ^1H NMR (300 MHz, $CDCl_3$): δ 0.85-0.9 (t, *J* = 7.5, 6.2 Hz, 3H), 1.1-2.4 (m, 12H), 2.26-2.42 (m, 1H), 3.43-3.60 (m, 1H), 4.0 (d, *J* = 3.3 Hz, 1H), 4.5 (d, *J*=12 Hz, 1H), 4.82-5.3(br s 1H excha. with D_2O), 4.9 (d, *J* = 12 Hz,1H), 7.25-7.37 (m, 5H). ^{13}C NMR (75 MHz, $CDCl_3$): δ 14.1, 22.6, 26.3, 26.7, 29.0, 29.7, 29.9, 31.7, 62.6, 73.2, 80.0, 127.2, 128.5, 136.5, 172.0. HRMS (ESI TOF) *m/z* calcd. for $C_{17}H_{27}N_3O_2Na$ $[M + Na]^+$ 342.1794; found 342.1793.

Compound 2a. To a solution of **10a** (0.3 g, 0.90 mmol) in dry methanol (5 mL) was added 10% Pd/C (0.04 g), and the solution was hydrogenated at 80 psi for 12 h. The catalyst was filtered, washed with methanol, and the filtrate was concentrated. The solvent was evaporated under reduced pressure, and the residue was subjected to ion-exchange chromatography (Dowex 50w × 8, 200–400 mesh) using 5% NH_4OH as the eluent. The amino acid **2a**, was obtained as a white solid and a free base (168 g, 80%). mp 219–220 °C (decomp), Lit.[1] mp 218–219 °C; $[\alpha]_D^{25}$ +5.6 (*c* .51, 1M HCl). Lit.[1] $[\alpha]_D^{22}$ +7.3 (*c* 0.37, 1M HCl). ^1H NMR (300 MHz, D_2O): δ 0.84 (t, *J* = 6.7 Hz), 3H, 1.19-1.45 (m, 10H), 1.50-1.83 (m, 2H), 3.38-3.50 (m, 1H), 4.08 (d, *J* = 3.8 Hz, 1H). ^{13}C NMR (75 MHz, D_2O): δ 14.0, 22.1, 24.7, 28.4, 28.7, 29.1, 31.2, 52.7, 69.3, 172.9; HRMS (ESI TOF) *m/z* calcd. for $C_{17}H_{28}O_3Na$ $[M + H]^+$ 204.1600; found 204.1604.

Compound 4b. Following the similar procedure described for **3a**, compound **4b** was prepared from **3b**. It was obtained as a colourless liquid **4b** in 92% yield. IR (neat) ν_{max}: 2108, 1635 cm^{-1}^{1}H NMR (300 MHz, $CDCl_3$): δ 0.92 (t, *J* = 6.9 Hz, 3H), 1.2-1.6 (m, 15H), 2.15-2.3 (m, 2H), 3.48 (dd, *J* = 8.8 Hz, *J* = 4.0 Hz 1H), 4.52 (t, *J* = 4.0 Hz, 1H), 4.68 (ABq, *J* = 12.6 Hz, 2H), 4.8 (t, *J* = 8.8 Hz, 1H), 5.24-5.38 (m, 1H), 5.62-5.8 (m, 2H), δ 7.22-7.4 (m, 5H). ^{13}C NMR (75 MHz, $CDCl_3$): δ 14.0, 22.4, 26.5, 27.0, 27.5, 29.2, 31.4, 71.9, 80.6, 85.2, 87.2, 105.5, 112.8, 127.6, 127.9, 128.0, 128.4, 133.8, 137.3. HRMS (ESI TOF) *m*/*z* calcd. for $C_{21}H_{30}O_4Na$ $[M + Na]^+$ 369.2144; found 369.2142.

Compound 5b. Following the similar procedure described for **5a**, compound **5b** was prepared from **4b**. It was obtained as a colourless liquid **5b** in 89% yield. R_f = 0.7 (pet ether/ethyl acetate 9.9:0.1); $[\alpha]_D^{32.4}$ +53.8 (*c* 0.12, $CHCl_3$); IR (neat) ν_{max}: 2924, 1460, 1016 cm^{-1}; ^{1}H NMR (300 MHz, $CDCl_3$): δ 0.8 (t, *J* = 6.9 Hz, 3H), 1.20-1.76 (m, 21H), 3.39-3.5 (dd, *J* = 9.0 Hz *J* = 4.5 Hz, 1H), 3.8- 4 (m, 1H), 4.54 (d, *J* = 9 Hz, 1H), 4.55 (d, *J* = 3.9 Hz, 1H), 4.8 (d, *J* = 9 Hz, 1H), 5.7 (d, *J*= 3.9 Hz 1H), 7.24-7.46 (m, 5H). ^{13}C NMR (75 MHz, $CDCl_3$): δ 14.0, 22.6, 26.5, 26.7, 29.1, 29.6, 31.7, 32.2, 72.0, 81.6, 103.8, 112.5, 127.9, 128.4, 128.4, 137.6. HRMS (ESI TOF) *m*/*z* calcd. for $C_{21}H_{32}O_4Na$ $[M + Na]^+$ 371.2198; found 371.2196.

Compound 6b. Following the similar procedure described for **6a**, compound **6b** was prepared from **5b**. It was obtained as a thick liquid in 72% yield. $[\alpha]_D^{33.5}$ +26 (*c* 0.24, $CHCl_3$); HRMS (ESI TOF) *m*/*z* calcd. for $C_{17}H_{28}O_3Na$ $[M + Na]^+$ 303.1936; found 303.1935; spectral data was found to be identical with **6a.**

Compound 7b. Following the similar procedure described for **7a**, compound **7b** was prepared from **6b**. It was obtained as a thick liquid in 93% yield. $[\alpha]_D^{33.7}$ +13.64 (*c* 0.52, $CHCl_3$); HRMS (ESI-TOF) *m*/*z* calcd. for $C_{33}H_{46}O_3SiNa$ $[M + Na]^+$ 541.3114; found 541.3112; spectral data was found to be identical with **7a.**

Compound 8b. Following the similar procedure described for **8a**, compound **8b** was prepared from **7b**. It was obtained as a thick liquid in 88% yield. $[\alpha]_D^{33.8}$ -49.56 (*c* 0.69, $CHCl_3$); HRMS (ESI TOF) m/z calcd. for $C_{33}H_{46}$ N_3O_2SiNa $[M + Na]^+$ 566.3179; found 566.3178; spectral data was found to be identical with **8a.**

Compound (9b). Following the similar procedure described for **9a**, compound **9b** was prepared from **8b**. It was obtained as a thick liquid in 98% yield. $[\alpha]_D^{33.9}$ -26 (*c* 0.67, $CHCl_3$); HRMS (ESI TOF) *m/z* calcd. for $C_{17}H_{27}N_3O_2Na$ $[M + Na]^+$ 328.2001; found 328.2000; spectral data was found to be identical with **9a.**

Compound (10b). Following the similar procedure described for **10a**, compound **10b** was prepared from **9b**. It was obtained as a thick liquid in 73% yield. $[\alpha]_D^{33.9}$ -21.5 (*c* 0.31, $CHCl_3$); HRMS (ESI TOF) *m/z* calcd. for $C_{17}H_{27}N_3O_2Na$ $[M + Na]^+$ 342.1794; found 342.1792; spectral data was found to be identical with **10a.**

Compound (2b). Following the similar procedure described for **2a**, compound **2b** was prepared from **10b**. It was obtained as a white solid. mp 220–222 °C, Lit.[2] mp 220–223 °C $[\alpha]_D^{30}$ -6.8 (*c* 0.51, 1M HCl). Lit.[2] $[\alpha]_D^{30}$ -6.2 (*c* 0.4, 1M HCl); HRMS (ESI TOF) *m/z* calcd. for $C_{17}H_{28}O_3Na$ $[M + H]^+$ 204.1600; found 204.1604; spectral data was found to be identical with **2a.**

References:

1. Wee, A. G. H.; McLeod, D. D. *J. Org. Chem.* **2003,** *68,* 6268–6273.
2. Shirode, N. M.; Deshmukh, A. R. A. S. *Tetrahedron* **2006,** *62,* 4615–4621.
