# Supplementary material for: Synthesis of (2S,3R)-3-amino-2-hydroxydecanoic acid and its enantiomer: a non-proteinogenic amino acid segment of the linear pentapeptide microginin
Source: Beilstein J Org Chem. 2014 Mar 17;10:667–71. doi: 10.3762/bjoc.10.59 (PMC3999816; doi:10.3762/bjoc.10.59)
Supplement: File 2 — Copies of NMR spectra. [file Beilstein_J_Org_Chem-10-667-s002.pdf]

Supporting Information

for

Synthesis of (2*S*,3*R*)-3-amino-2-hydroxydecanoic acid and its enantiomer: a non-proteinogenic amino acid segment of the linear pentapeptide microginin

Rajendra S. Rohokale and Dilip D. Dhavale*

Address: Department of Chemistry, Garware Research Centre, University of Pune, Pune - 411 007, India

Email: Dilip D. Dhavale - ddd@chem.unipune.ac.in

* Corresponding author

Copies of NMR spectra

^{1}H NMR spectrum of compound **4a** S3

^{1}H NMR spectrum of compound **4b** S5

^{1}H NMR spectrum of compound **5a** S7

^{1}H NMR spectrum of compound **5b**S9

^{1}H NMR spectrum of compound **6a** S11

^{1}H NMR spectrum of compound **7a** S13

^{1}H NMR spectrum of compound **8a** S15

^{1}H NMR spectrum of compound **9a** S17

^{1}H NMR spectrum of compound **10a** S19

^{1}H NMR spectrum of compound **(+)-2a** S21

^{13}C NMR spectrum of compound **4a**S6

^{13}C NMR spectrum of compound **4b** S8

^{13}C NMR spectrum of compound **5a** S10

^{13}C NMR spectrum of compound **5b**S12

^{13}C NMR spectrum of compound **6a** S14

^{13}C NMR spectrum of compound **7a** S16

^{13}C NMR spectrum of compound **8a** S18

^{13}C NMR spectrum of compound **9a** S20

^{13}C NMR spectrum of compound **10a** S22

^{13}C NMR spectrum of compound **(+)-2a** S24

^{1}H NMR and ^{13}C NMR Spectra

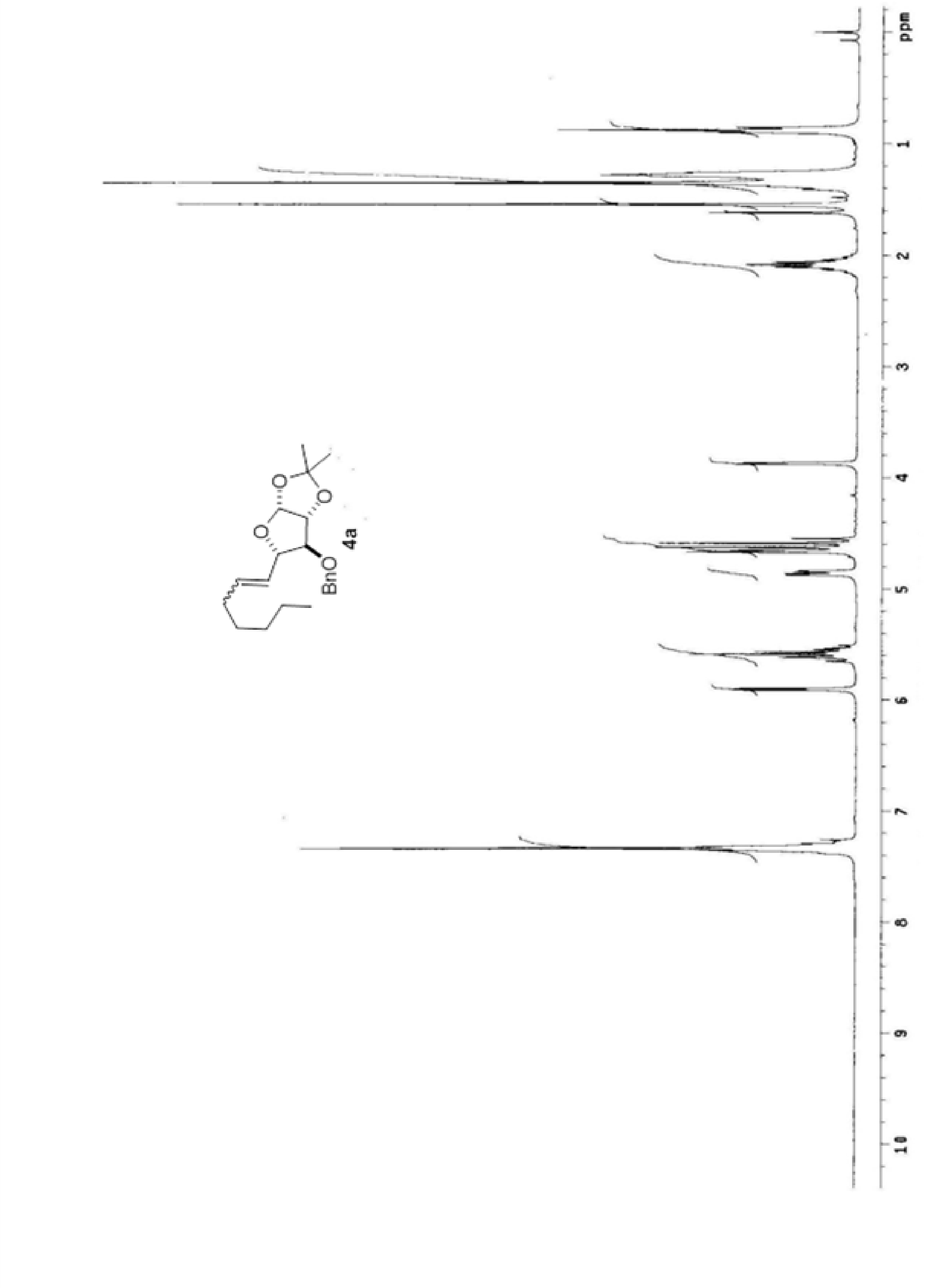

Figure 1: ^{1}H NMR (300 MHz, $CDCl_3$) Spectrum of Compound **4a**.

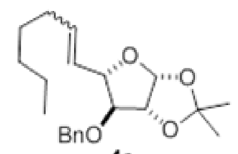

4a

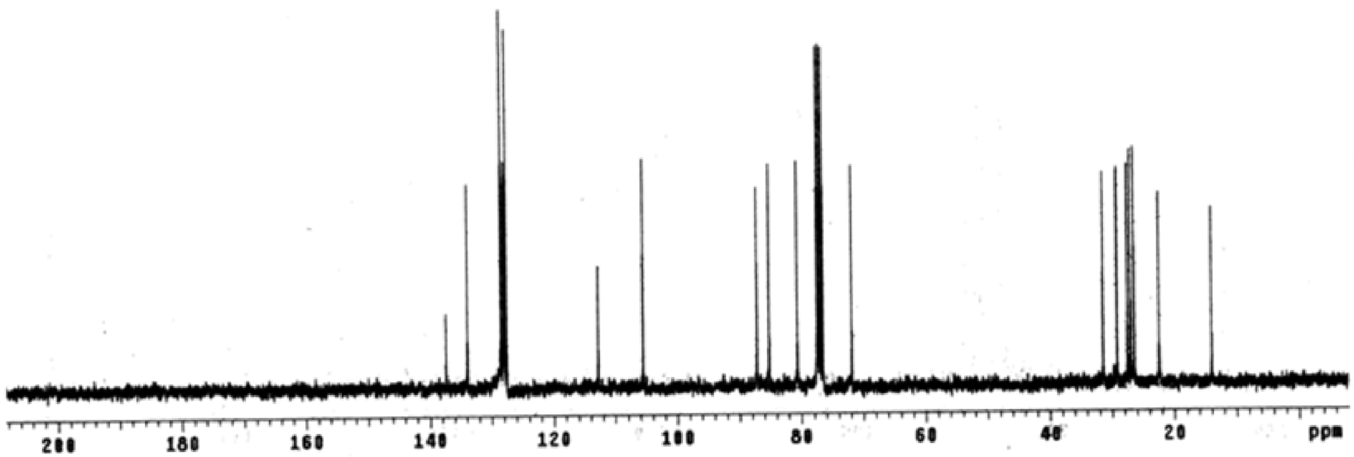

Figure 2: ^{13}C NMR (300 MHz, $CDCl_3$) Spectrum of Compound **4a**.

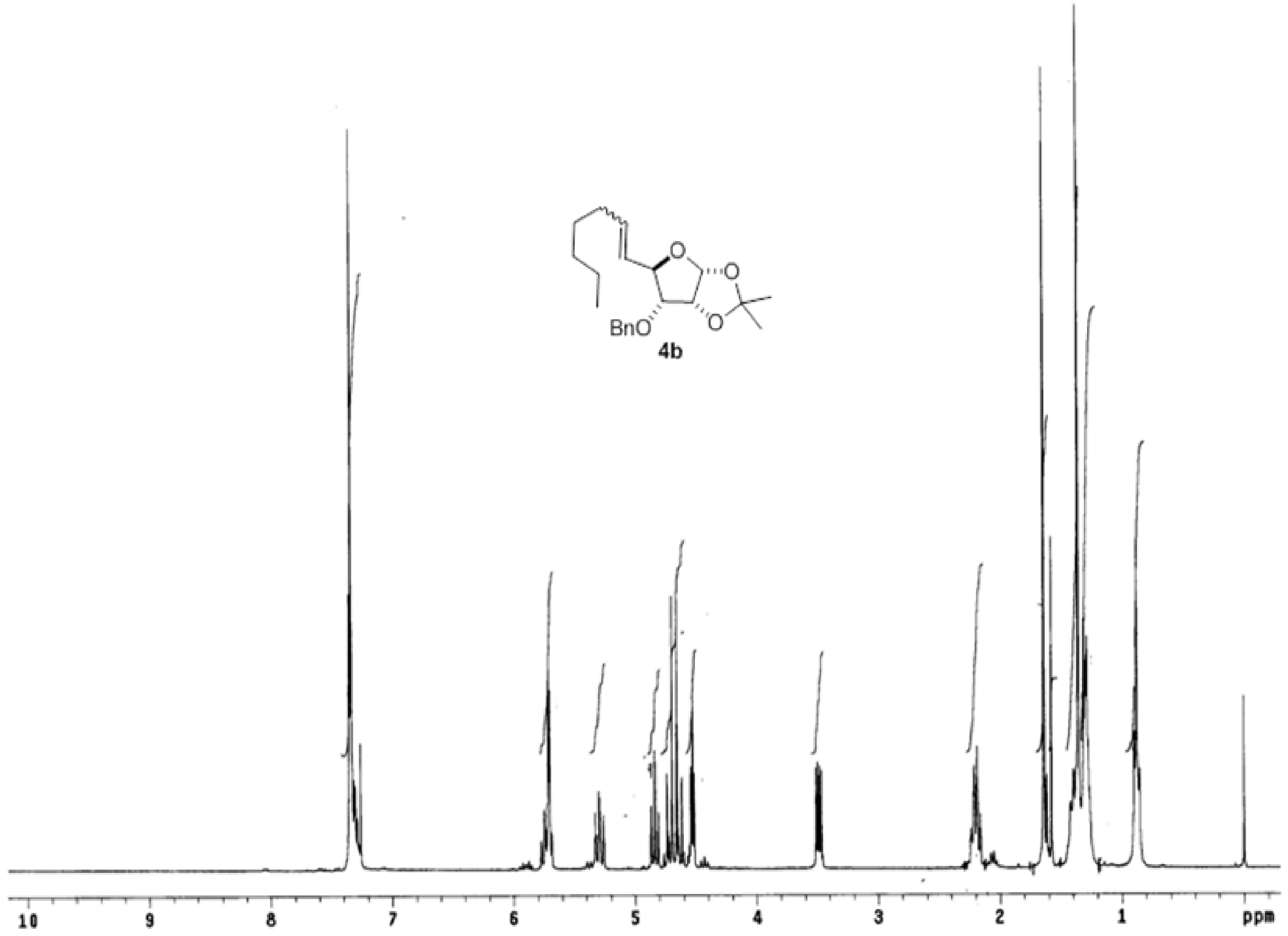

Figure 3: ^{1}H NMR (300 MHz, $CDCl_3$) Spectrum of Compound **4b**.

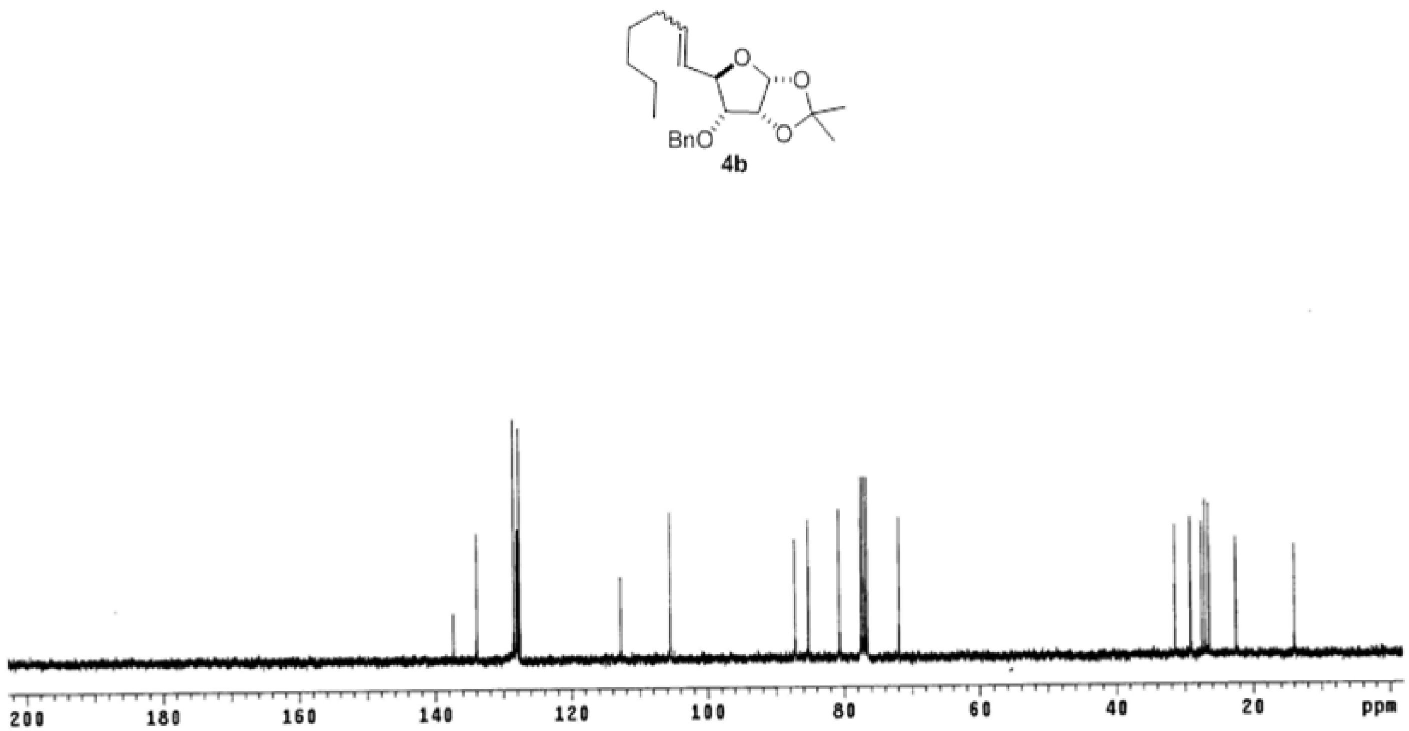

Figure 4: ^{13}C NMR (300 MHz, $CDCl_3$) Spectrum of Compound **4b**.

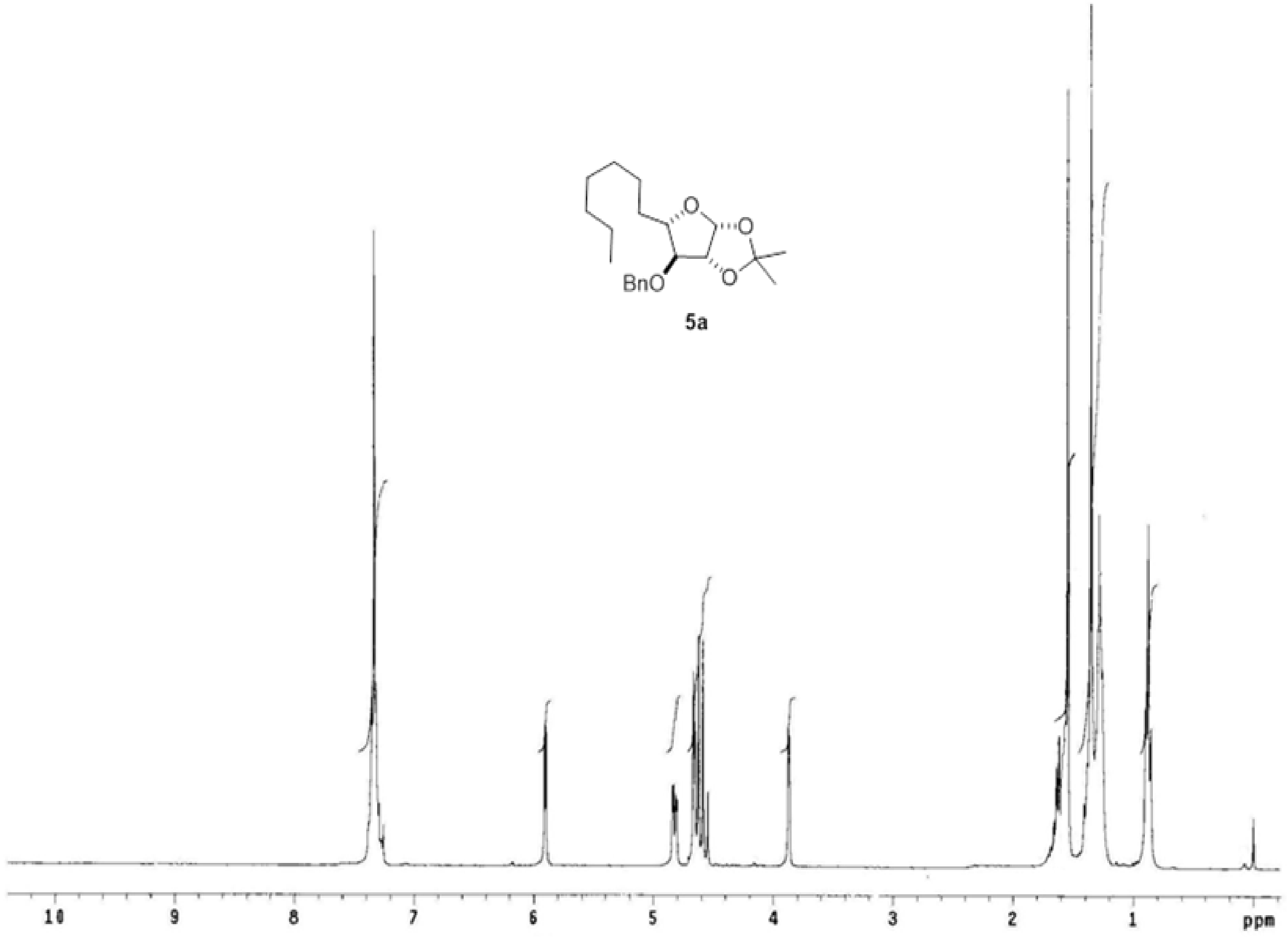

Figure 5: ^{1}H NMR (300 MHz, $CDCl_3$) Spectrum of Compound **5a**.

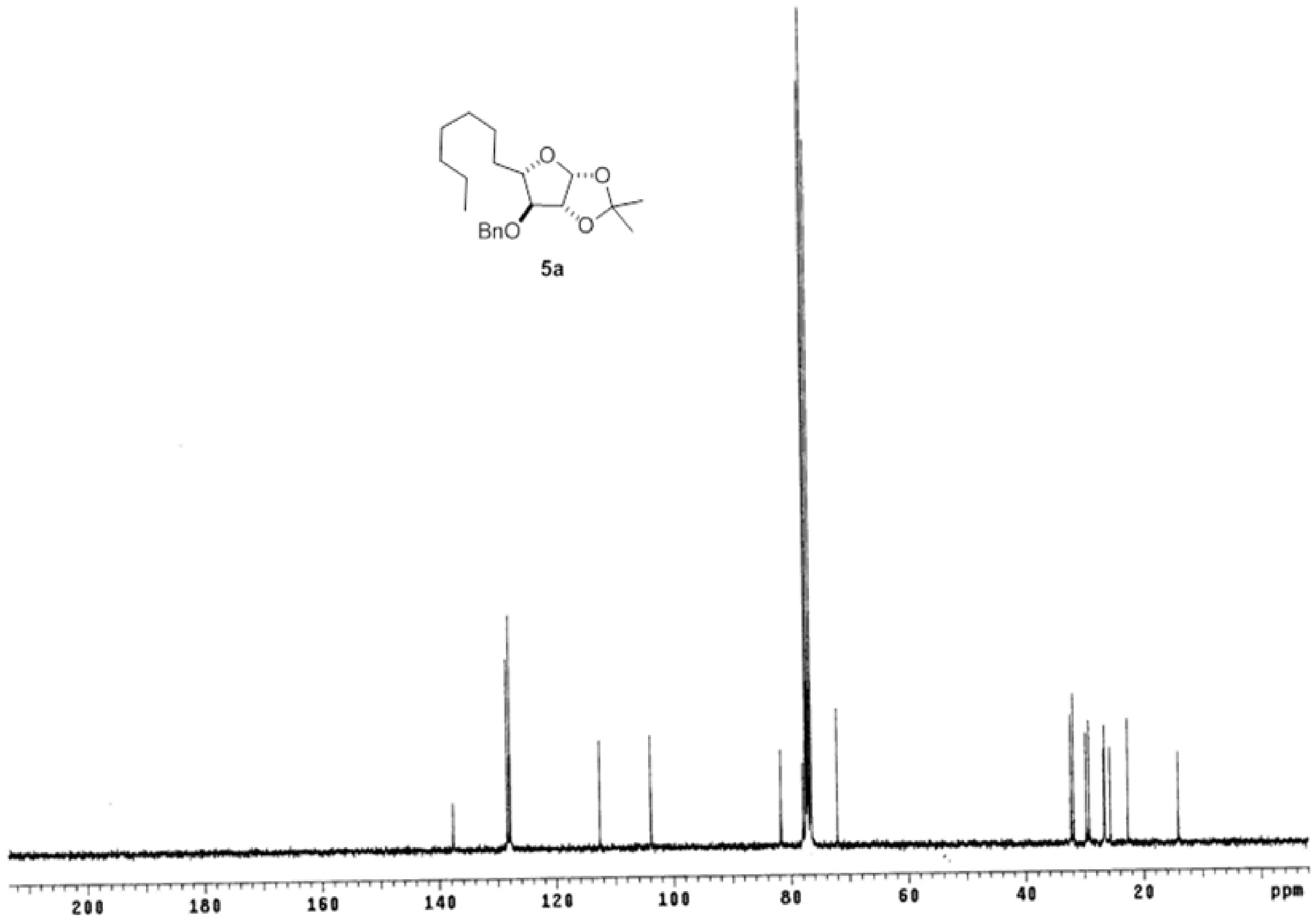

Figure 6: ^{13}C NMR (300 MHz, $CDCl_3$) Spectrum of Compound **5a**.

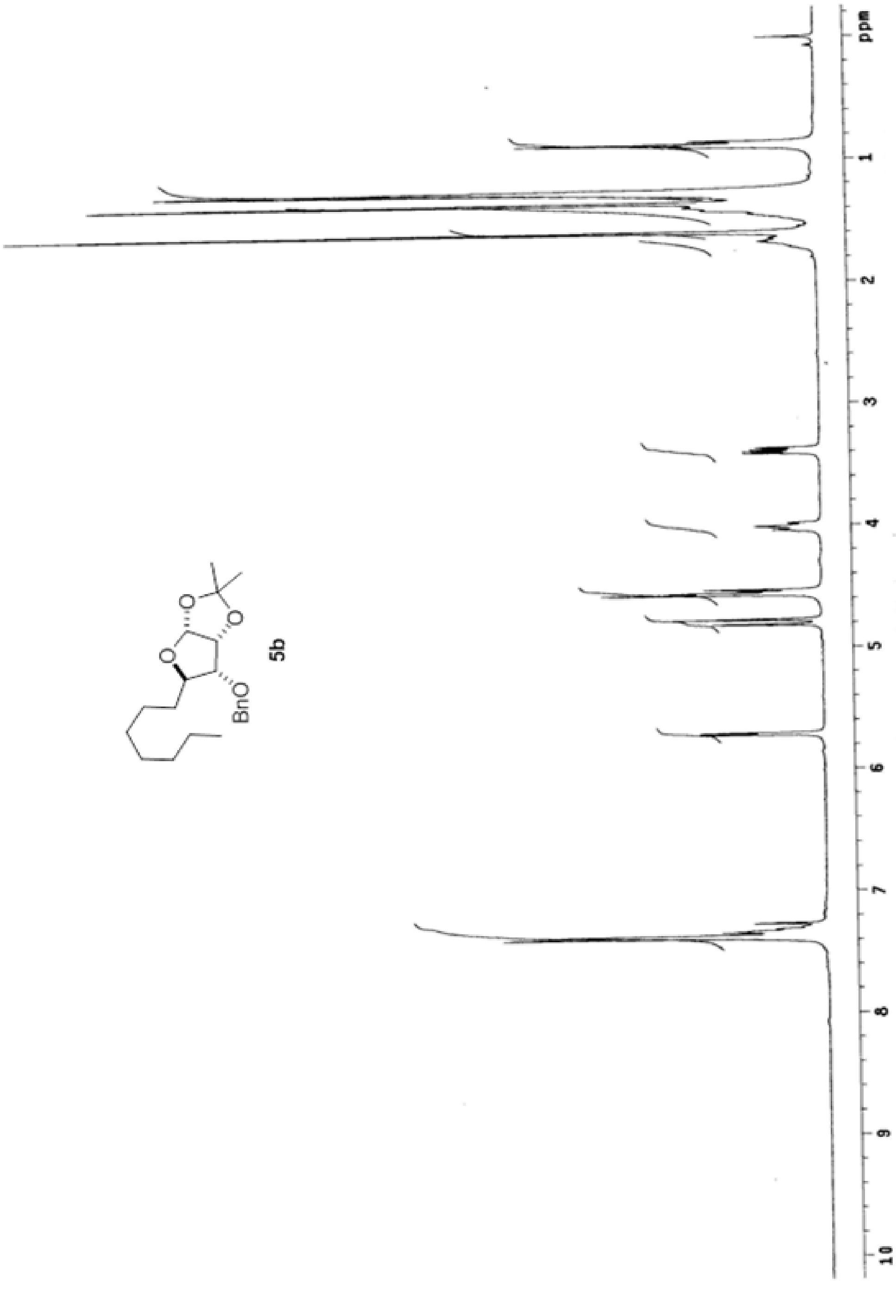

Figure 7: ^1H NMR (300 MHz, $CDCl_3$) Spectrum of Compound **5b**.

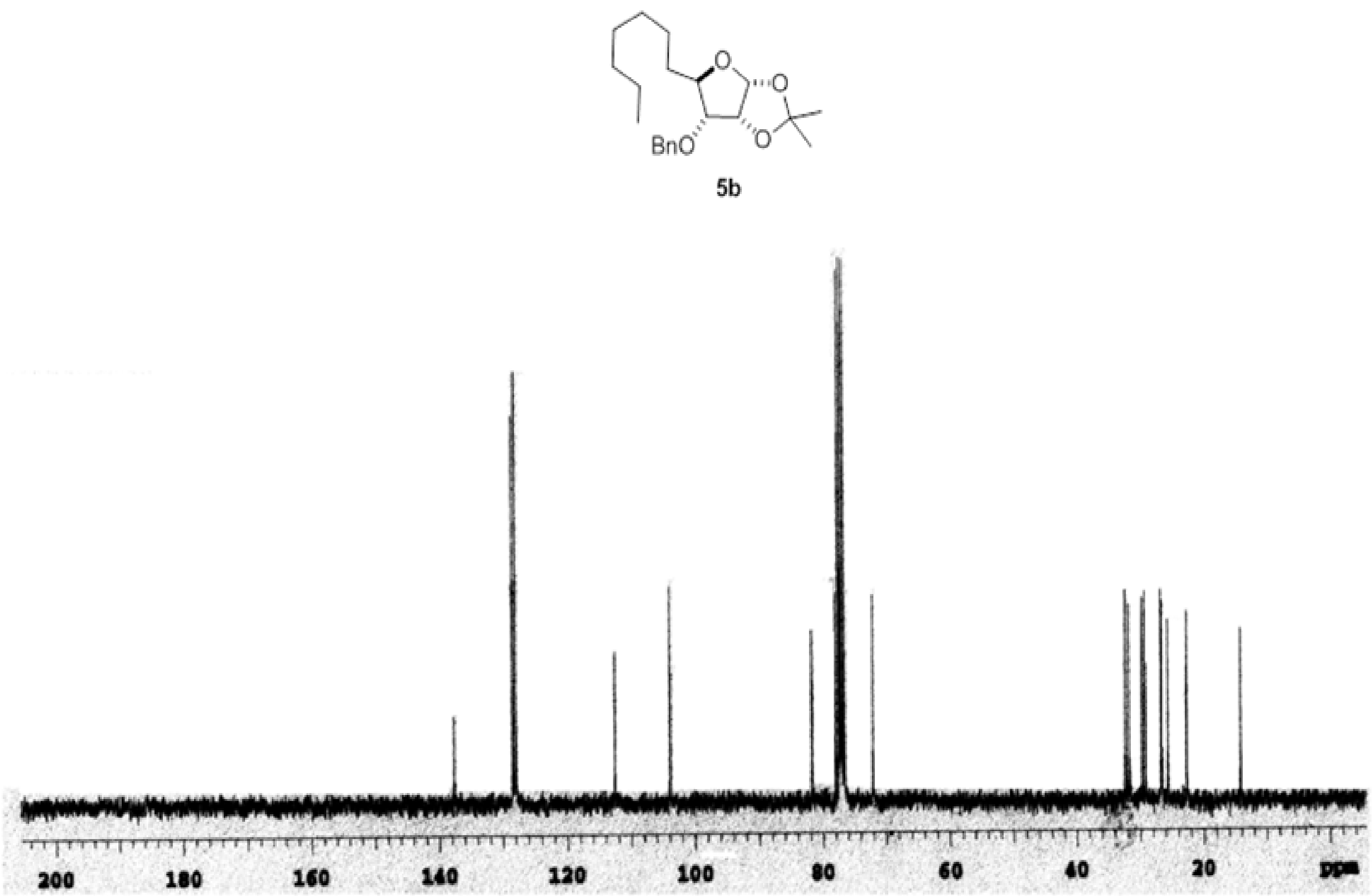

5b

Figure 8: ^{13}C NMR (300 MHz, $CDCl_3$) Spectrum of Compound **5b**.

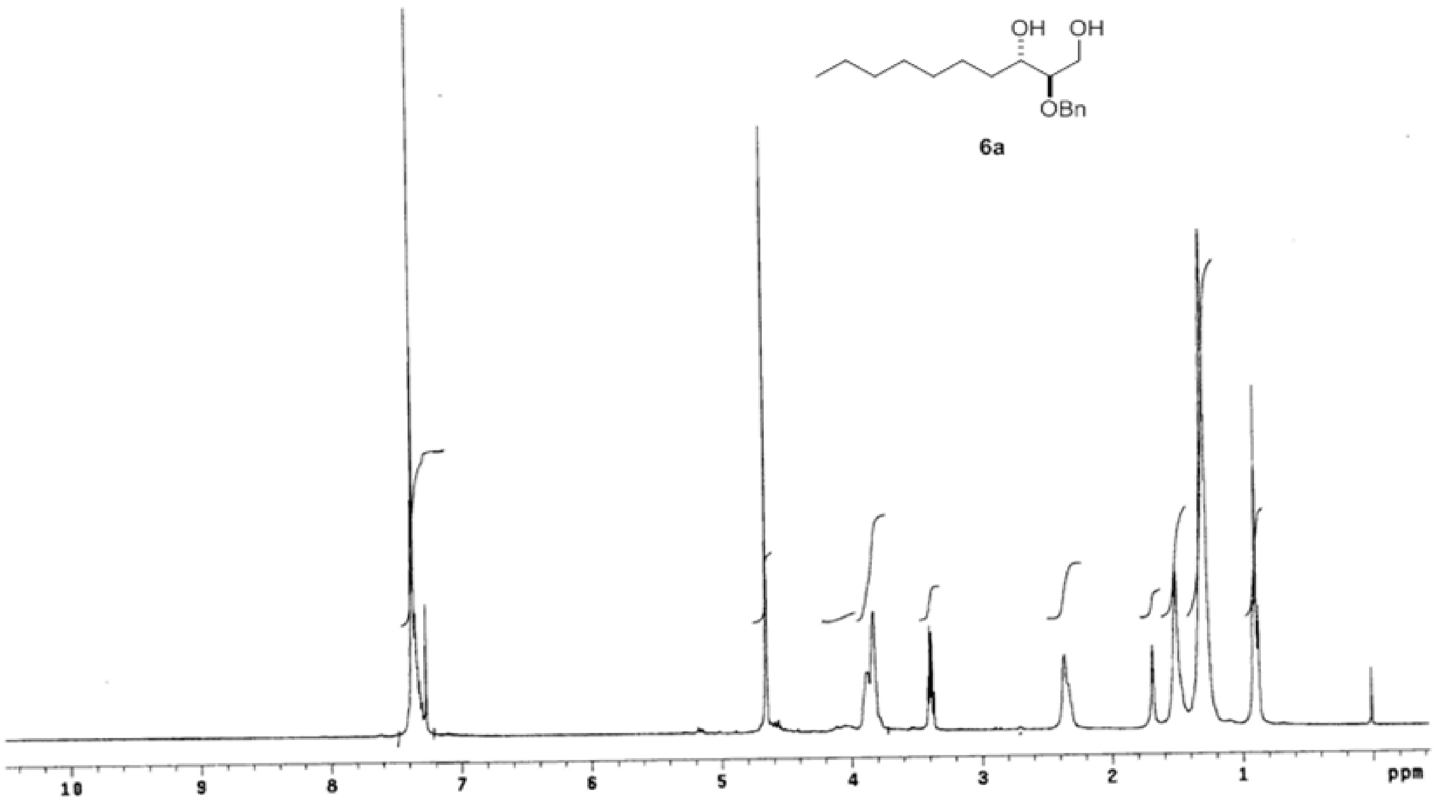

Figure 9: ^{1}H NMR (300 MHz, $CDCl_3$) Spectrum of Compound **6a.**

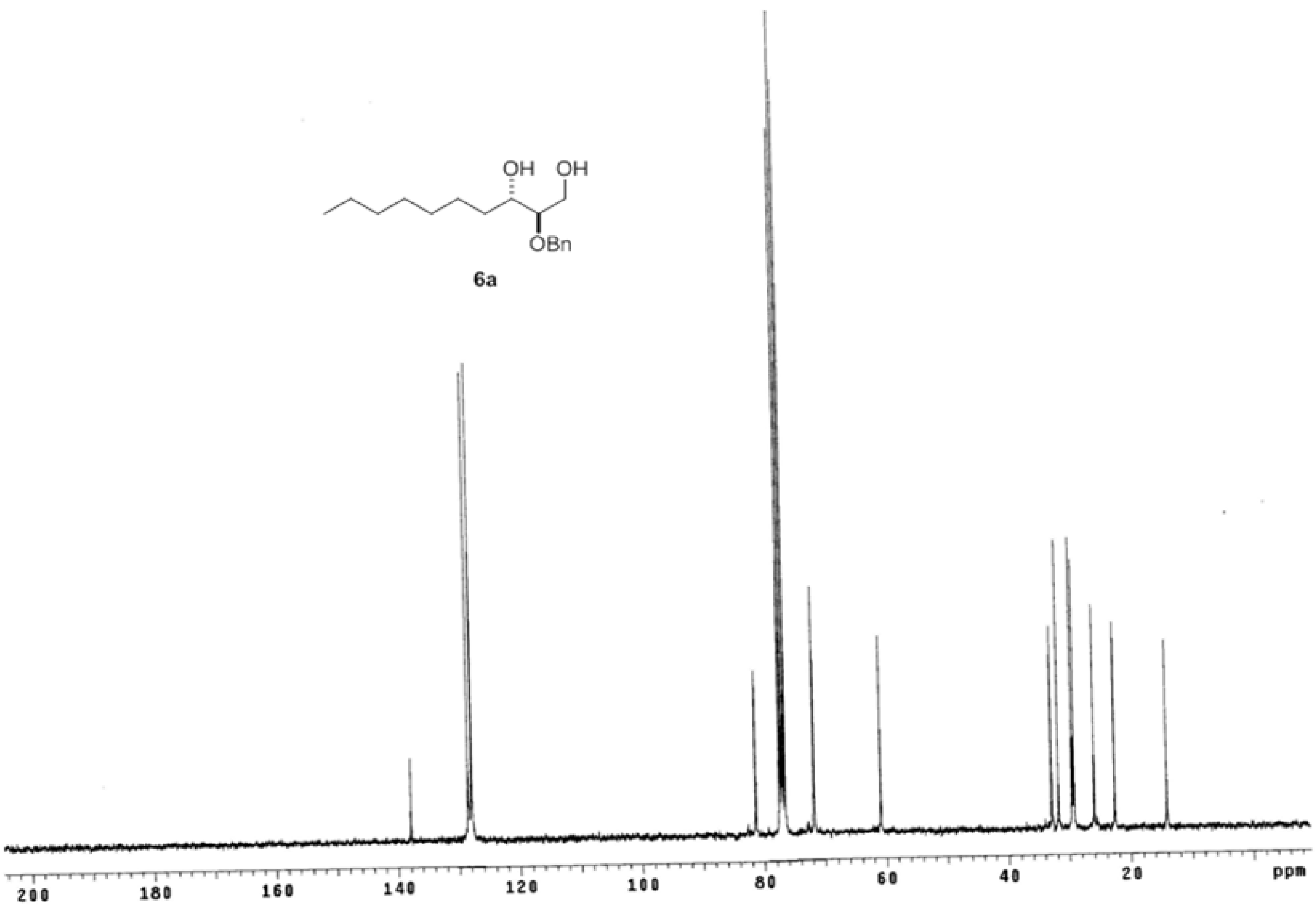

Figure 10: ^{13}C NMR (300 MHz, $CDCl_3$) Spectrum of Compound **6a**.

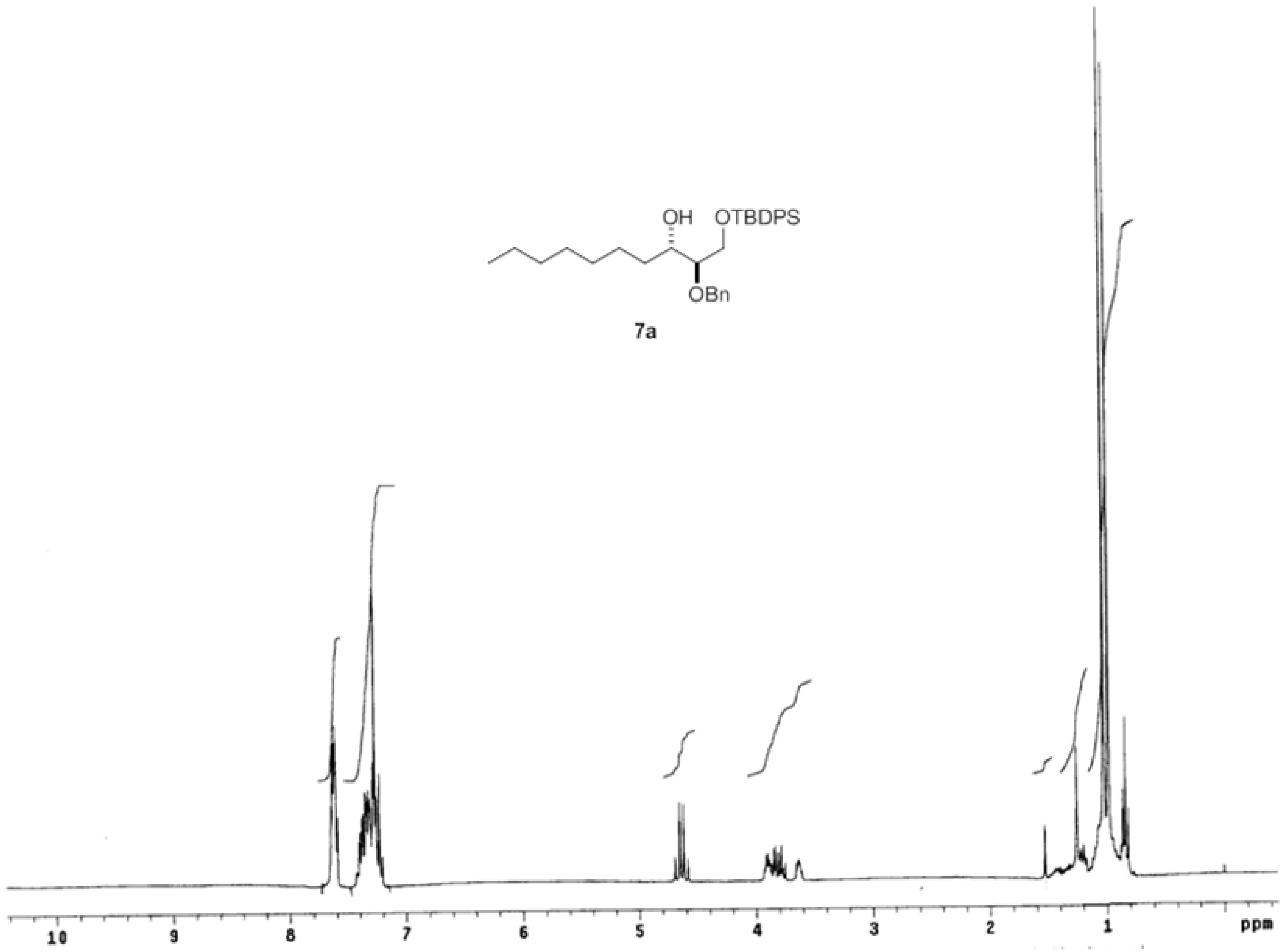

Figure 11: ^{1}H NMR (300 MHz, $CDCl_3$) Spectrum of Compound **7a**.

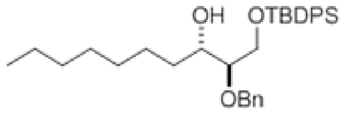

7a

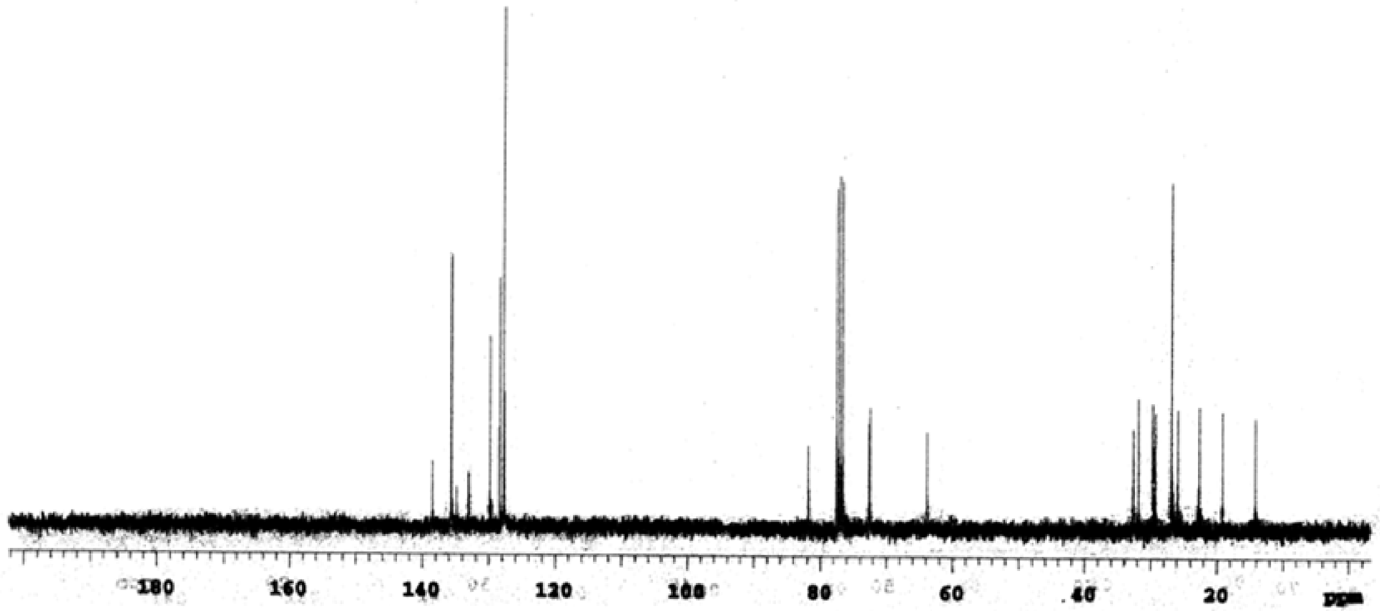

Figure 12: ^{13}C NMR (300 MHz, $CDCl_3$) Spectrum of Compound **7a**.

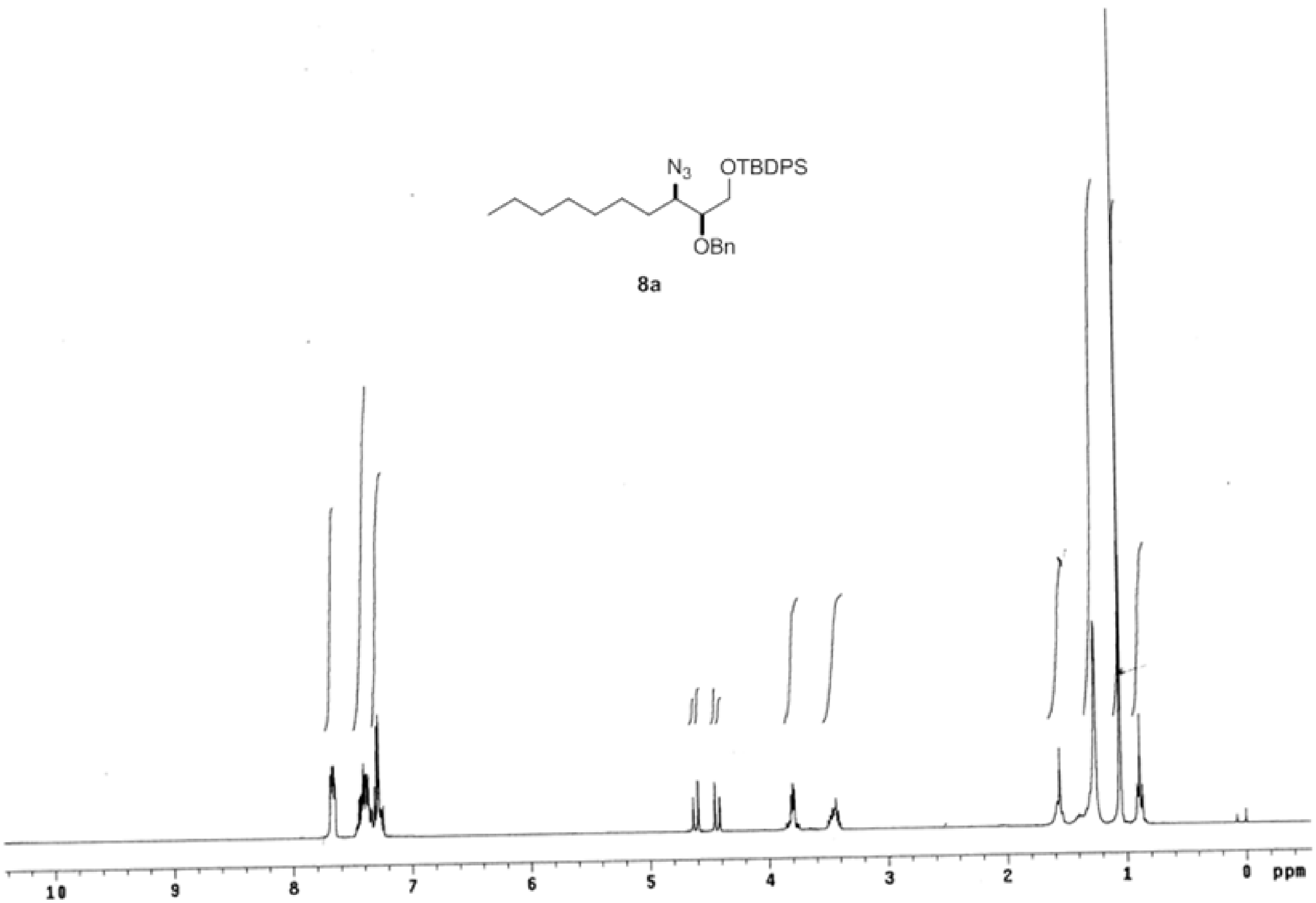

Figure 13: ^{1}H NMR (300 MHz, $CDCl_3$) Spectrum of Compound **8a**.

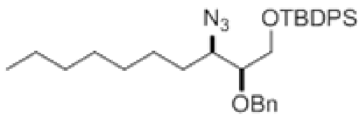

8a

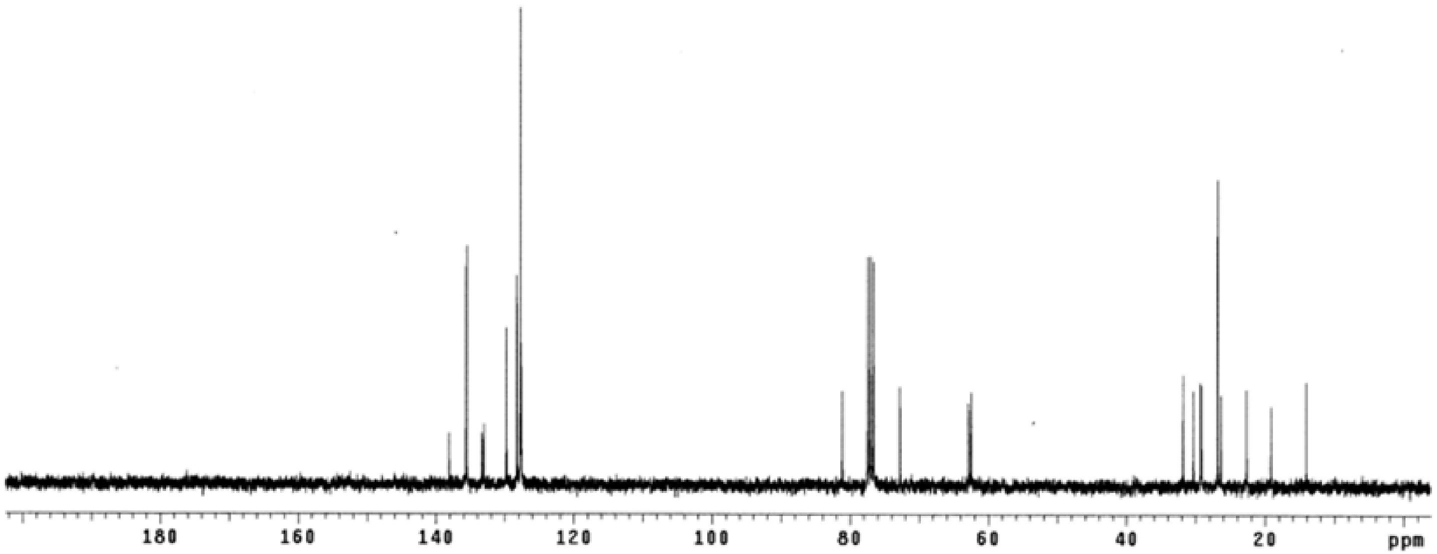

Figure 14: ^{13}C NMR (300 MHz, $CDCl_3$) Spectrum of Compound **8a.**

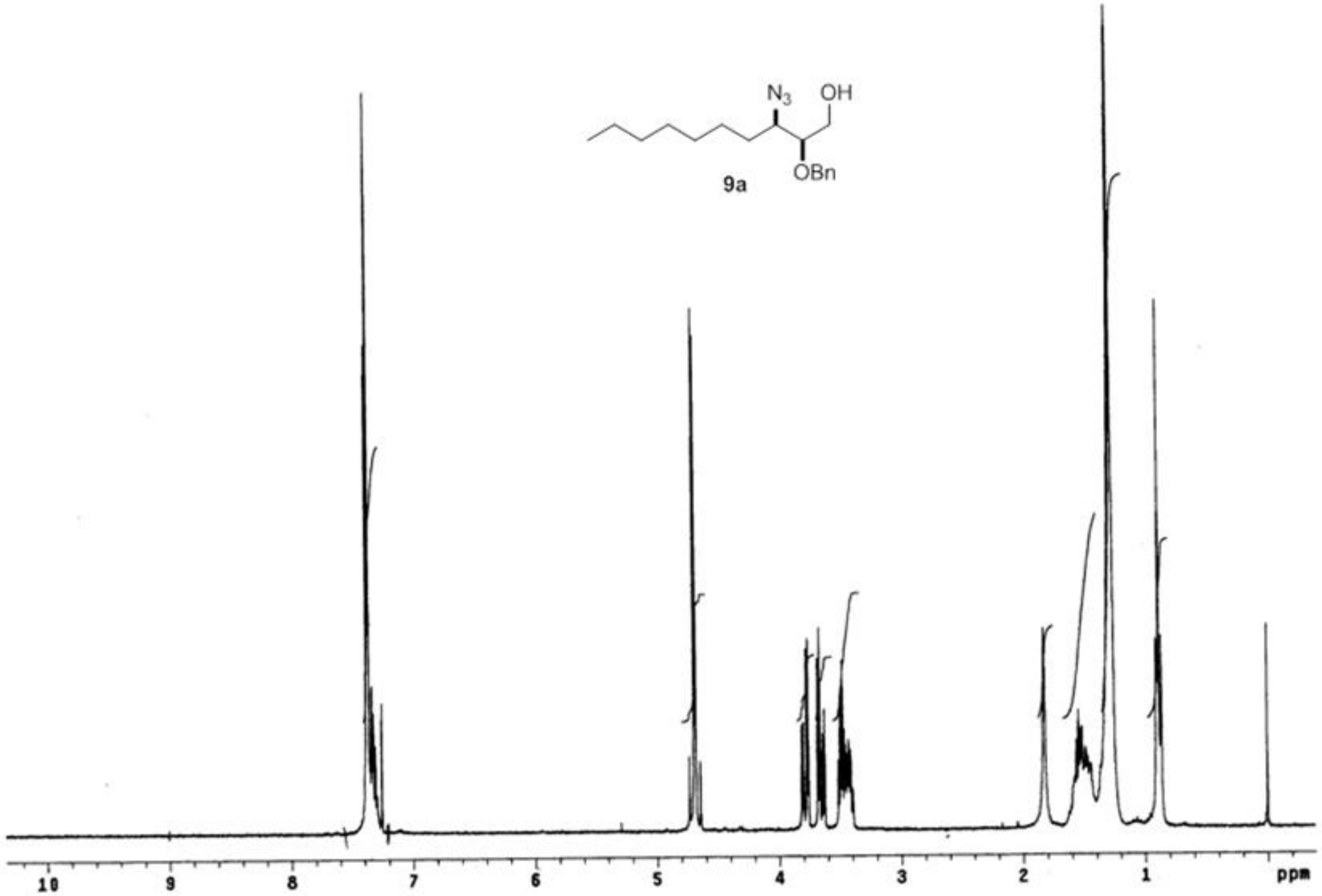

Figure 15: ^{1}H NMR (300 MHz, $CDCl_3$) Spectrum of Compound **9a**.

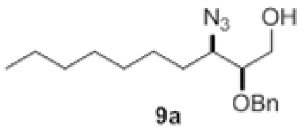

9a

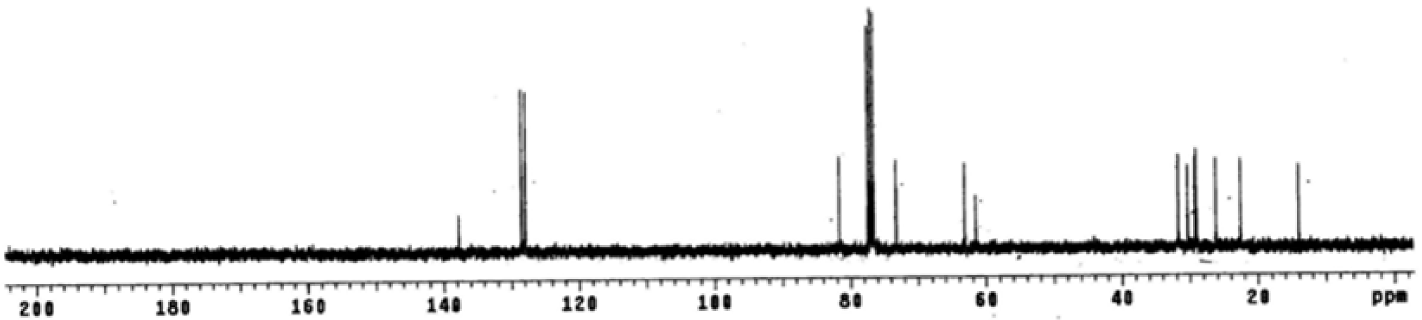

Figure 16: ^{13}C NMR (300 MHz, $CDCl_3$) Spectrum of Compound **9a**.

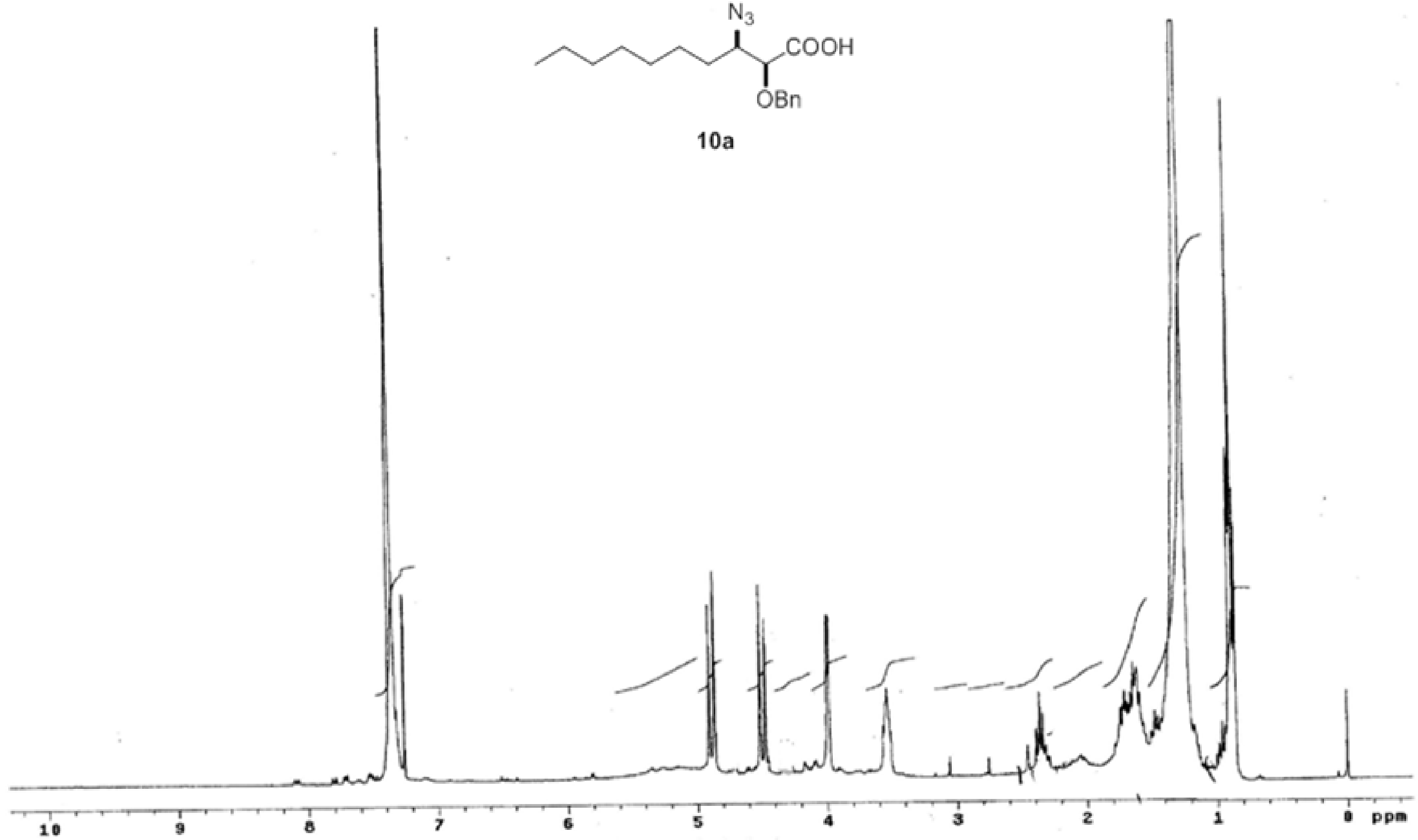

Figure 17: ^{1}H NMR (300 MHz, $CDCl_3$) Spectrum of Compound **10a**.

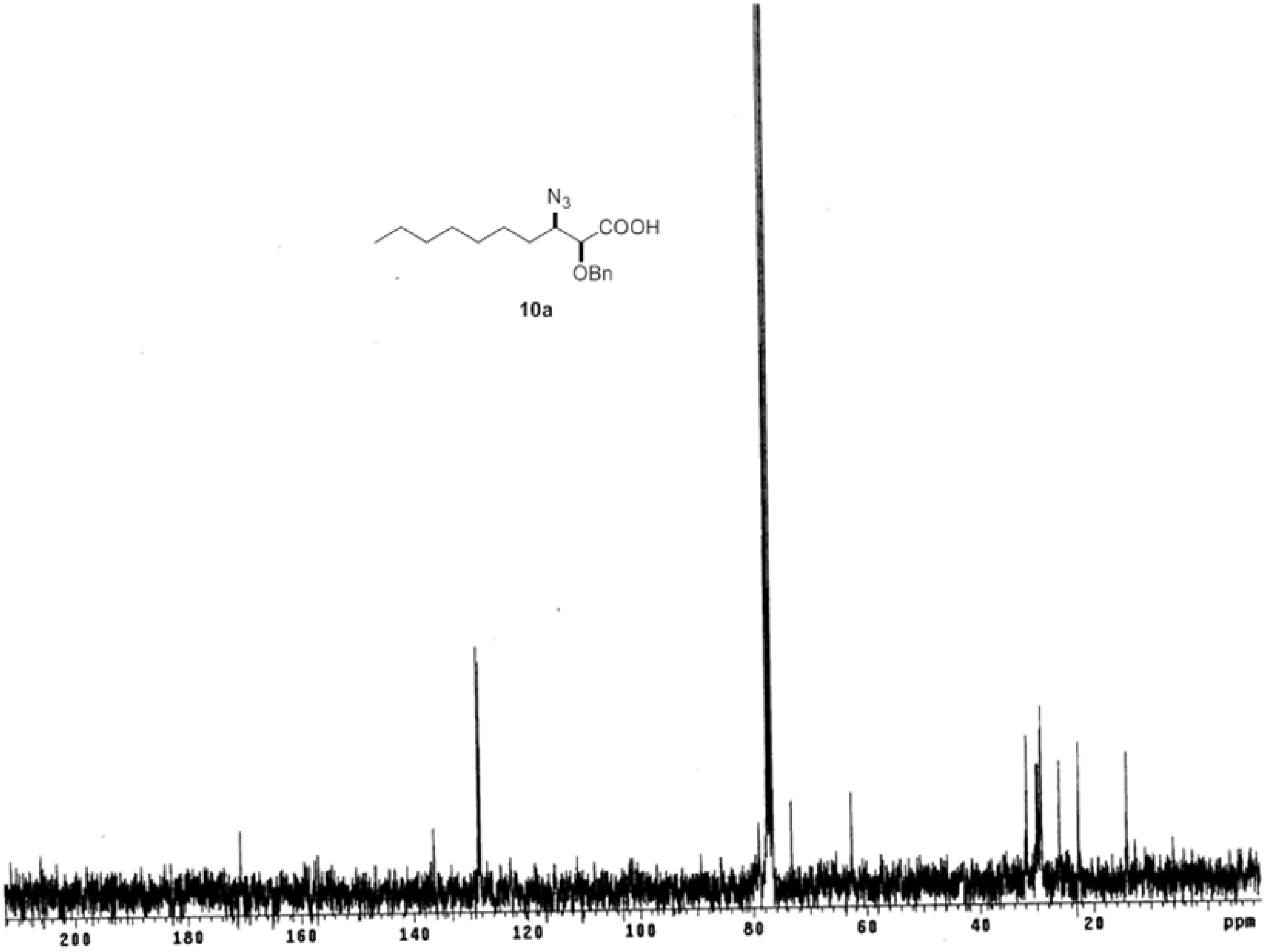

Figure 18: ^{13}C NMR (300 MHz, $CDCl_3$) Spectrum of Compound **10a**.

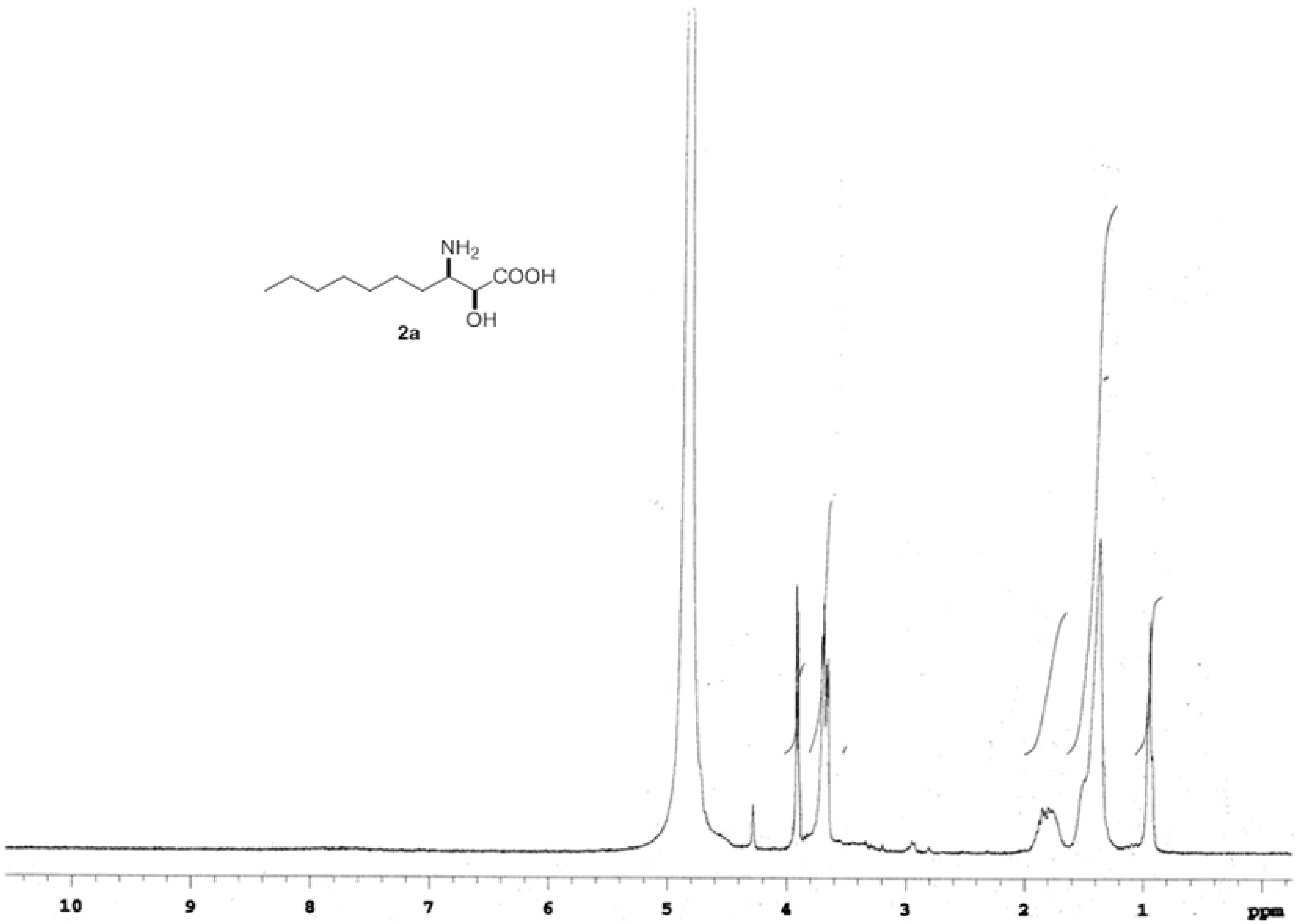

Figure 18: ^1H NMR (300 MHz, D_2O) Spectrum of Compound **2a**.

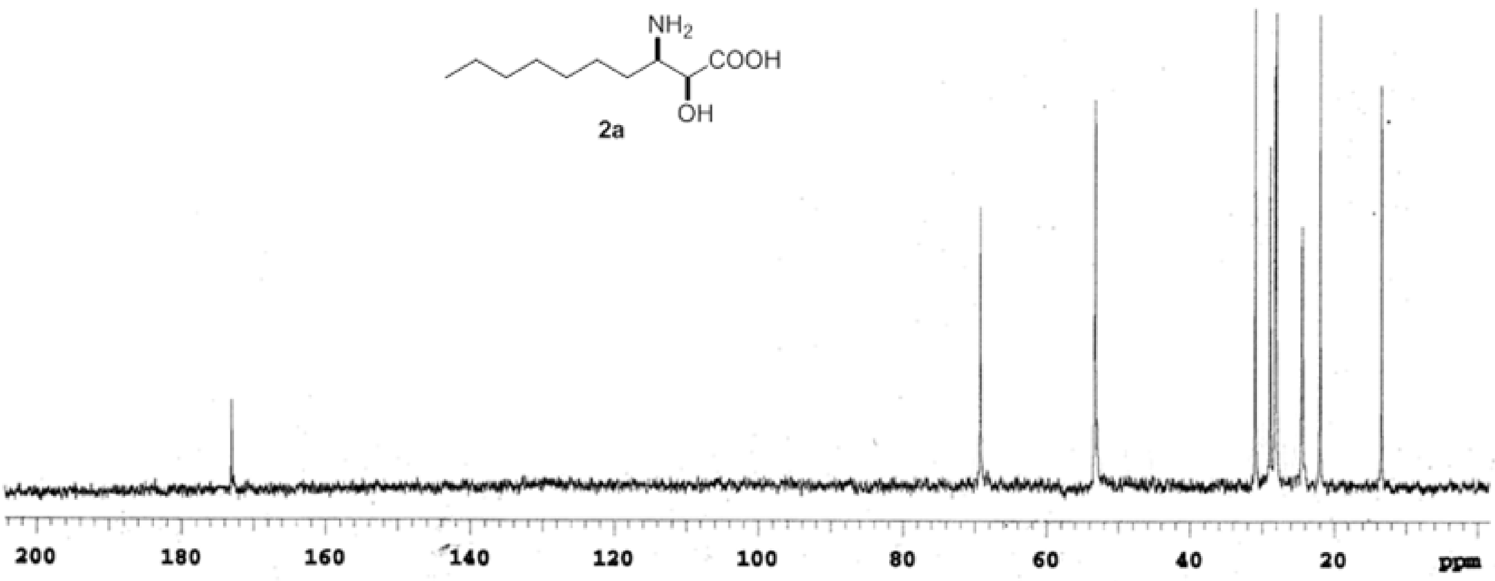

Figure 20: ^{13}C NMR (300 MHz, D_2O) Spectrum of Compound **2a**.
